# CryoGrid-PIXUL-RNA: high throughput RNA isolation platform for tissue transcript analysis

**DOI:** 10.1186/s12864-023-09527-7

**Published:** 2023-08-08

**Authors:** Scott A. Schactler, Stephen J. Scheuerman, Andrea Lius, William A. Altemeier, Dowon An, Thomas J. Matula, Michal Mikula, Maria Kulecka, Oleg Denisenko, Daniel Mar, Karol Bomsztyk

**Affiliations:** 1 UW Medicine South Lake Union, University of Washington, Seattle, WA 98109 USA; 2 Institute for Stem Cell and Regenerative Medicine, University of Washington, Seattle, WA 98109 USA; 3 Center for Lung Biology, University of Washington, Seattle, WA 98109 USA; 4 Center for Industrial and Medical Ultrasound, Applied Physics Laboratory, University of Washington, Seattle, WA 98195 USA; 5 Department of Genetics, Maria Sklodowska-Curie National Research Institute of Oncology, 02-781 Warsaw, Poland; 6 Department of Gastroenterology, Hepatology and Clinical Oncology, Centre for Postgraduate Medical Education, 01-813 Warsaw, Poland; 7 Matchstick Technologies, Inc, Kirkland, WA 98033 USA

**Keywords:** CryoGrid for tissues cryostoring and sampling, Proteinase K, PIXUL RNA extraction, RNA sequencing, Transcriptomics

## Abstract

**Background:**

Disease molecular complexity requires high throughput workflows to map disease pathways through analysis of vast tissue repositories. Great progress has been made in tissue multiomics analytical technologies. To match the high throughput of these advanced analytical platforms, we have previously developed a multipurpose 96-well microplate sonicator, PIXUL, that can be used in multiple workflows to extract analytes from cultured cells and tissue fragments for various downstream molecular assays. And yet, the sample preparation devices, such as PIXUL, along with the downstream multiomics analytical capabilities have not been fully exploited to interrogate tissues because storing and sampling of such biospecimens remain, in comparison, inefficient.

**Results:**

To mitigate this tissue interrogation bottleneck, we have developed a low-cost user-friendly system, CryoGrid, to catalog, cryostore and sample tissue fragments. TRIzol is widely used to isolate RNA but it is labor-intensive, hazardous, requires fume-hoods, and is an expensive reagent. Columns are also commonly used to extract RNA but they involve many steps, are prone to human errors, and are also expensive. Both TRIzol and column protocols use test tubes. We developed a microplate PIXUL-based TRIzol-free and column-free RNA isolation protocol that uses a buffer containing proteinase K (PK buffer). We have integrated the CryoGrid system with PIXUL-based PK buffer, TRIzol, and PureLink column methods to isolate RNA for gene-specific qPCR and genome-wide transcript analyses. CryoGrid-PIXUL, when integrated with either PK buffer, TRIzol or PureLink column RNA isolation protocols, yielded similar transcript profiles in frozen organs (brain, heart, kidney and liver) from a mouse model of sepsis.

**Conclusions:**

RNA isolation using the CryoGrid-PIXUL system combined with the 96-well microplate PK buffer method offers an inexpensive user-friendly high throughput workflow to study transcriptional responses in tissues in health and disease as well as in therapeutic interventions.

**Supplementary Information:**

The online version contains supplementary material available at 10.1186/s12864-023-09527-7.

## Background

We have previously developed a 96-well microplate sonicator, PIXUL, where an array of miniature piezoelectric transducers generate high-intensity focused ultrasound (HIFU) in each of the 96 microplate wells thereby homogenizing and/or fragmenting contents in each well. This instrument offers unparalleled sample preparation throughput capabilities for a broad range of high throughput analytical applications [1–3]. In sharp contrast, available tissue storing and sampling tools lack the throughput to fully exploit the PIXUL sample preparation capabilities to interrogate solid biospecimens.

Freezing is a common way to preserve tissues for storage and transport [4–6]. Typically, tissues are snap-frozen using either liquid nitrogen or dry ice/isopentane and stored in vials. Space shortages in deep freezers (-80^o^C) are a recurring problem for many labs, especially those that process hundreds of tissue samples. Unfortunately, and not infrequently, older samples are discarded to make room for new samples. Laboratory freezers may contain thousands of samples in tubes marked with handwritten or printed numbers, dates, and sample types. Tubes are stored in small cardboard boxes that are also labeled and are either stored loose or are kept in sliding drawers in freezer racks. Without a map guided by codes, finding specific samples can be a challenge and typically involves pulling the racks out one at a time until the needed box is found.

Biopsy needles have been used for decades to examine tissue pathology. These are designed for soft tissues, and their through length is 10-20 mm. As such, traditional biopsy devices are not suitable to sample frozen tissues that are hard. There are also punch needles that could be used to sample frozen tissue, but it is difficult to get consistent core sizes and to remove frozen cores from the needle’s tip. For a host of molecular analyses and histology, it would be advantageous for researchers, as well as clinical labs, to cryostore solid tissue specimens in such a way that the same piece of frozen tissue is suitable for multiple samplings, without thawing.

There are many different methods to isolate nucleic acids; the first one was pioneered by Friedrich Miescher more than 150 years ago [7]. The RNA isolation methods often use guanidinium and other biohazardous materials [8–11). TRIzol is one of the most commonly used reagents to isolate RNA [10, 11] but the protocol is labor-intensive, relatively slow and uses hazardous solvents requiring working in safety hoods. Further, TRIzol reagent is expensive and involves costly material shipment. Various columns (e.g. PureLink) to purify RNA are also widely used but they involve many steps, are prone to human errors, and are also expensive [10, 11]. Proteinase K offers simple, microplate-based and biohazard-free ways to extract nucleic acids from tissues [1, 12–17].

Here, to match the high throughput sample preparation power of PIXUL [2, 3, 18] and downstream multiomics analytical methods [17, 19], the CryoGrid system was developed for cryostoring (CryoTray) and sampling (CryoCore) tissues. Using QR-coded CryoTrays, Google Drive and an iPAD the CryoGrid system was integrated with the PIXUL [1–3] and with a new microplate proteinase K (PK)-based buffer as well as the established TRIzol and PureLink column tissue RNA extraction methods for RNA-qPCR and RNA-seq analyses.

## Materials, devices and methods

Hardware/labware (Table S1) and kits/enzymes (Table S2) catalog numbers and commercial suppliers are listed in the supplementary tables.

### Reagents

TRIzol (Life Technologies 15,596,018). O.C.T. compound (Fisher Scientific 4585). Dithiothreitol (DTT, D0632), EDTA (E3134), Tris–HCl (T3253) were from Sigma. Sodium chloride (NaCl S-271-3) and Triton X-100 (BP151) from Fisher Scientific. NP40 (198,596) from MP Biomedicals. Dulbecco’s Modified Eagle Medium (DMEM SH30021.0) from HyClone, penicillin/streptomycin (P/S 15,749) from Invitrogen, fetal bovine serum (FBS 43635-500) from Jr. Scientific, and phosphate buffered saline (PBS 70013-032), Chloroform (J.T. Baker, 9180-01), Ethanol (Decon Labs, 2716), and Isopropanol (Acros Organics, 3223-0010).

### Buffers

Preparations of all buffers and stock solutions were done with nuclease free reagents and ultrapure distilled RNAse/DNAse free H_2_O. PBS: 137 mM NaCl, 10 mM Sodium phosphate, 2.7 mM KCl, pH 7.4; TE: 10 mM Tris-HCl, 1 mM EDTA, pH 7.5; Immunoprecipitation (IP) buffer: 150 mM NaCl, 50 mM Tris–HCl (pH 7.5), 5 mM EDTA, NP-40 (0.5% vol/vol), Triton X-100 (1.0% vol/vol); Elution buffer-Proteinase K: 25mM Tris Base, 1% IP Buffer, 1mM EDTA, 80 µg/ml Proteinase K; Proteinase K buffer (PK buffer): 10mM Tris-HCl pH 8.0, 10mM EDTA, 0.5% SDS, 500 µg/ml Proteinase K, and 40mM DTT. PureLink RNA Micro Kit buffers prepared as directed and Lysis Buffer prepared using DTT as optioned in the kit’s preparation protocol.

### Devices

#### CryoGrid system

**Fig. 1:**
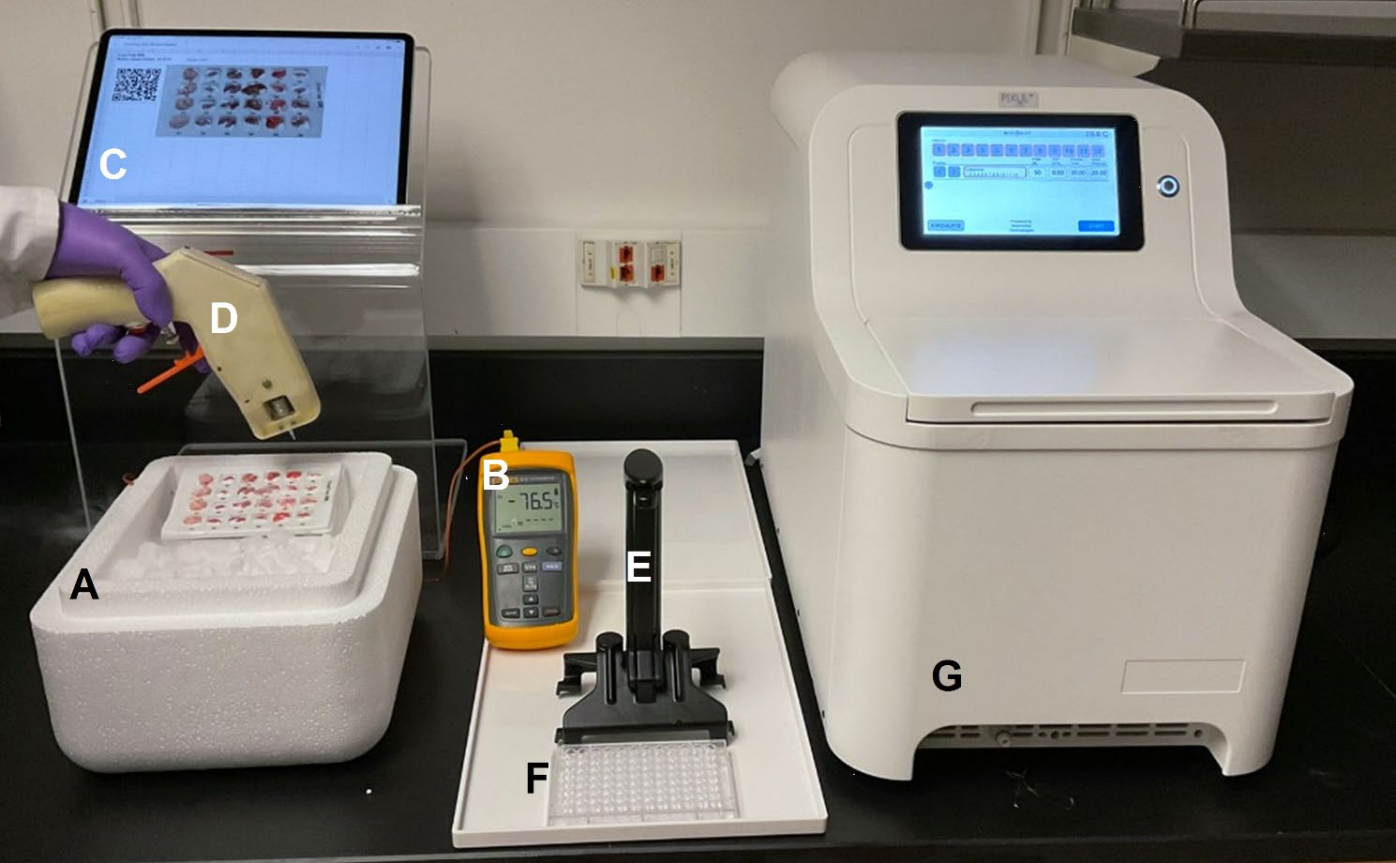
CryoGrid-PIXUL system. ***A***, CryoTray (with QR code recorded online in Google Drive, see Fig. 1S) is seated in a CryoBlock cooled in an off-the-shelf Styrofoam box containing dry ice pellets (CryoBox)(< -70^o^C). Tissue pieces are placed in CryoTray pockets following a pre-designed experimental template in Google Drive. Tissues are kept frozen and immobilized by adding optimal cutting temperature (OCT) or CryoGel cryogenic media. CryoTray-embedded frozen tissue layout is then photographed (iPad) and uploaded online to the matching QR-coded Google Sheet containing relevant metadata. ***B***, Thermometer thermocouple probe wire is threaded through a hole in the side of a CryoBox and inserted into an aperture on the side of the CryoBlock to monitor the temperature. ***C***, iPad, on an acrylic stand, displays the CryoTray layout of annotated tissues, and provides the means to read or hear recorded notes and to type-in and/or dictate comments using voice recording in online Google Sheet. ***D***, CryoCore is a motor-driven miniature hole-saw tool to extract cores of frozen tissues embedded in CryoGrid and then eject the cores directly into wells of 96-well PIXUL plates. ***E***, PlateHandle is a tool to facilitate the transfer of 96-well plates in-and-out of PIXUL. ***F***, 96-well plate for sonicating samples in PIXUL. ***G***, PIXUL instrument

The complexity and heterogeneity of disease pathways require analysis of large numbers of tissue samples. Almost any disease, and often therapeutic intervention, are systemic conditions where animal models provide the means to understanding multiorgan dysfunction. To store and sample large numbers of biospecimens for multiomics analysis, we designed a platform for freezing and cryostoring multiple tissue samples and engineered a hand-held rotary tool for rapid sampling of frozen tissues (Fig. 1 and S1-S2). This system, which we call the CryoGrid, consists of CryoBox, CryoBlock, thermometer/thermocouple, QR barcoded CryoTrays and CryoCore. The CryoBox is a Styrofoam box filled with dry ice pellets. There is a small hole in the wall of the box to pass a thermocouple wire. CryoBlock, machined from aluminum, with 24 (6 × 4) cube-sized pockets in the top surface to accommodate 6 × 4 CryoTray (Fig.S1), is seated in the dry-ice-containing CryoBox. The top surface of the CryoBlock is tilted 30^o^ to optimize ergonomics. The CryoBlock has a small aperture on the side, close to the block top, to insert the tip of the thermocouple probe to monitor the block’s freezing and maintenance temperature. CryoBlock chilled in the CryoBox (<-70^o^C) is used to freeze tissues in the CryoTrays and/or keep the samples frozen while extracting cores.

CryoTrays are heat-molded from polystyrene sheets into rectangular trays that contain an array (6 × 4 wells) of round corner cube-shaped (1 × 1 × 1 cm) pockets that serve as receptacles for freezing and storing tissues (Figs. S1-S2). CryoTrays are covered with a transparent polystyrene lid that has a QR code that uniquely identifies a given CryoTray. The QR code is entered into a web-based Google Drive storage database allowing users to enter, edit, and share metadata of the tissues frozen in each QR-coded CryoTray. The CryoTrays with frozen tissues identified with a QR code are stored in a deep freezer in standard pull-out aluminum drawers at -80^o^C. An upright deep freezer can store > 100,000 ~ 1gm frozen tissue fragments in 24-well CryoTrays.

To engineer a novel-design hand-held battery-powered rotary tool for rapid sampling of frozen tissues, CryoCore (Fig. 1 and S1), we used the idea of a trephine (from Greek word trypanon, an instrument for boring). With CryoCore, the same frozen tissue can be sampled multiple times without thawing, yielding reproducible core sizes (~ 1-2mm^3^ or ~ 1–2 mg). The CryoCore design was tailored for multisampling tissues stored in CryoTrays (Fig. S2). The estimated (by 6pg DNA content/cell [20]) number of cells/tissue core are as follows: brain ~ 0.4 e + 06; heart ~ 0.3 e + 06; kidney ~ 1.2 e + 06 and liver ~ 0.9 e + 06.

#### PIXUL

A 96-well plate sample preparation sonicator for multiomics applications (Matchstick Technologies, Inc, Kirkland, WA and Active Motif, Carlsbad, CA) [1].

## Methods

### Cell lines and treatment

Human kidney HEK293 cell lines were grown in DMEM supplemented with glutamine, penicillin, streptomycin, and 10% fetal bovine serum in round-bottom 96-well polystyrene plates at density ~ 200,000 cells per well. For time-point experiments, cells were serum-deprived (0.1% FBS) overnight and at specific time points were treated with either 10% FBS or PBS as we described previously [1].

### Cell line RNA isolation using PIXUL

Proteins with high binding activity for ribonucleases (RNases), such as the commercially available recombinant RNaseOUT (Table S2), potently inhibit RNases [21]. Serum-deprived HEK293 cells were treated with 10% serum for 0, 30, 60, and 120 min, supernatants were aspirated, and 100ul of either elution buffer-Proteinase K with and without RNaseOUT (1U/20ul buffer), or TRIzol was added to individual wells, and plates were sonicated for 4 min in PIXUL with settings: Pulse = 50, PRF = 1.0 Burst = 20. The elution buffer-Proteinase K samples were then transferred to 500ul microcentrifuge tubes, boiled for 10 min in a water bath to inactivate Proteinase K, put on ice and then centrifuged for 15 min at 16,000 g and 4 ^o^C, and finally the supernatants were collected in new 500ul microcentrifuge tubes. TRIzol samples were used to purify RNA using the protocol described in the below ‘RNA Isolation’ section. Contaminating genomic DNA (gDNA) in RNA extraction is a challenge, especially for analysis of rare transcripts requiring the use of deoxyribonucleases (DNases) to remove even minor amounts of gDNA [22, 23]. To assess the effects of contaminating gDNA, the RNA prepared using each protocol was treated with and without DNase I (Table S2) as described below in ‘DNase I Treatment’ and then used in reverse transcriptase (RT) reaction as described below in ‘Matrix quantitative reverse transcription real time PCR (Matrix RT-qPCR)’ to generate cDNAs as templates in real-time (RT-qPCR) with primers to serum-inducible and housekeeping genes listed in Table S3.

### Frozen organs from a mouse model of sepsis

Mouse organs were used from female 8–12-week-old C57bl/6 mice that were injected intraperitoneally (IP) with 5 mg/kg lipopolysaccharide (LPS) in 200 µl PBS or with 200 µl PBS as control. After 12 h mice were euthanized by isoflurane overdose and confirmatory cervical dislocation. Post mortem brains, hearts, kidneys and livers were harvested and frozen.

### Tissue freezing, sampling, jetting cores into PIXUL plate, and sonicating

Before freezing tissues, a 24-well CryoTray is placed into the chilled CryoBlock maintained at < -70^o^C in a CryoBox filled with dry ice pellets. Fresh tissues are immediately put on ice and then one by one are placed in individual pockets of the 24-well CryoTray with a small amount of embedding matrix (e.g., OCT or Leica CryoGel) injected into the bottom of the wells for rapid freezing and immobilizing of tissue fragments (Fig. 1). The total amount of time to freeze 24 tissues is less than 20 min. Frozen tissues are placed in the wells and immobilized with either OCT or Leica CrypGel. The CryoTray, with tissues are covered by a QR code labeled lid, is stored at -80^o^C.

For sampling, the CryoTray with frozen tissues is inserted in a chilled CryoBlock (< -70^o^C) in a CryoBox filled with dry ice pellets (Fig. 1). An iPad is used to display a Google Sheet document with the organ layout legend and convenient access to metadata to facilitate sampling with the CryoCore. Before coring, the CryoCore trephine is cooled by plunging it in the dry ice pellets. CryoCore tissue cores are jetted with PBS (drawn from the CryoCore syringe reservoir) directly into wells of a 96-well round-bottom heat-resistant polypropylene PIXUL plate kept on ice. The 96-well plate is covered with a MicroAmp Optical Adhesive tape and a small “V” is cut in the top of each well to allow jetting samples into the wells while preventing splashing and cross-contamination. One or two cores per well are sampled from each tissue fragment. After collecting all the samples, the plate is covered with an additional optical adhesive tape, centrifuged for 30 s at 500xg, the optical adhesive tapes are then removed carefully and discarded, and the PBS buffer from CryoCore sample ejection is aspirated from the wells. 100 µl of extraction buffer (PK buffer, TRIzol, or Lysis Buffer for PureLink Micro RNA Kit (Table S2)) is added to the wells, and the 96-well plate is covered with a new optical adhesive film and placed into the PIXUL sonicator. Samples are processed for 30 s in PIXUL with settings: Pulse = 50, PRF = 1.0 Burst = 20. After sonication, plates are centrifuged (30 s at 500xg) to collect debris at the bottom of the wells.

### RNA isolation

#### PK buffer samples

After PIXUL treatment, samples are immediately incubated in the same polypropylene PIXUL plate at 95 °C for 20 min, and then put on ice for 5 min. Cooled samples are centrifuged in a plate centrifuge for 10 min at 4,200xg and 4^o^C and then put back on ice. 60 µl of clear supernatant (without any floating residue) from each well is carefully transferred via pipette to a new semi-skirted 96-well PCR plate on ice. Isolation of nucleic acids with 1.8x SPRI beads is done as per the Omega Bio-tek protocol (Norcross, GA) with a two-minute final SPRI bead drying time and final elution to 50 µl of ultrapure ddH_2_O, and then samples are put on ice. Finally, DNase I and RNAseOut are added to the eluted RNA samples and DNase I digestion is done as written below.

#### TRIzol samples

After PIXUL treatment, TRIzol samples are moved under the hood to individual 500 µl microcentrifuge tubes with an additional 100 µl of TRizol (total = 200 µl) and taken through the TRIzol RNA extraction protocol (ThermoFisher/Invitrogen, Waltham, MA) following steps 3–8 of “Lyse samples and separate phases” followed by steps 1–4 of “Isolate RNA” with a final elution volume of 50 µl ultrapure ddH_2_O followed by DNase I digestion as written below.

#### PureLink column protocol

After PIXUL treatment, each sample is transferred to its own 500ul microcentrifuge tube with an additional 250ul Lysis Buffer and centrifuged at 12,000xg for 2 min at room temperature. The supernatant was transferred to a new 500 µl tube and then followed the manufacturer’s protocol (ThermoFisher/Invitrogen, Waltham, MA) at step 1 of the ‘Binding, Washing, and Elution’ section of ‘Purifying RNA from Animal Tissues’ using on-column DNase digestion and a final elution volume of 20µl.

### DNase I treatment

DNase I enzyme used at a ratio of 1U of DNase I per 1 µg of estimated maximum possible sample contaminating DNA. DNase I is diluted 1:10 with ultrapure ddH_2_O and then mixed at a 1:1 ratio with DNase I 10X Reaction Buffer. 10 µl of the combined DNase I/Buffer mixture is added to each sample and incubated at at 37 °C for 10 min in an Eppendorf ThermoMixer® C to digest contaminating DNA. For the PK buffer protocol, 40U RNaseOUT per 20 µl RNA solution and DTT to a final concentration of 5 mM are added to each sample prior to the 37 °C digestion. Finally, 100mM EDTA is added to each sample to a final concentration of 5 mM EDTA and then incubated at 75 °C for 10 min to inactivate the DNase I enzyme.

### Matrix quantitative reverse transcription real time PCR (Matrix RT-qPCR)

Isolated RNA (100ng) was reverse transcribed with Superscript, 0.2 mM dNTP (GeneScript, 95040-880), and random hexamers in 10 µl reactions in 96-well microplates for 10 min at 50 °C then 10 min at 80 °C. RT reactions were diluted 10-fold with elution buffer prior to running qPCR. Housekeeping genes were used to normalize qPCR results [24]. RT-qPCR primers are listed in supplementary Table S3. We used our previously developed software, PCRCrunch, to acquire, store and analyze qPCR data sets generated by Matrix RT-qPCR [25].

### mRNA-sequencing (RNA-seq)

After isolation, RNA was run through Zymo RNA Clean & Concentrator. Sequencing libraries were prepared using Zymo-Seq RiboFree Total RNA Library Kit with RNA between 205-480ng and libraries amplified between 13 and 14 cycles of PCR as per manufacturer’s protocol. Quality of libraries was assessed by Agilent 4200 TapeStation system, qPCR with organ- and sepsis-specific primers (to show retained specificity) and Collibri Library Quantification Kit. Libraries were diluted as per Illumina protocol to a final pooled loading concentration of 650pM in resuspension buffer (RSB) plus Tween 20 with a 10% PhiX spike-in and sequenced in Illumina P2 cartridges on NextSeq 2000 that employed a dual-index, paired-end, 61 base read length (PE61).

Quality control was done for all sequencing fastq.gz files with FastQC [26]. Visualization of read coverage was done using the RSeQC ‘geneBody_coverage.py’ function with default settings [27]. Transcript integrity number (TIN) was derived using the RSeQC ‘TIN.py’ function with default settings [28]. FastQC and RSeQC figures and data were compiled for visualization with MultiQC [29] and PCRCrunch.

Reads were mapped to the NCBI Genome Reference Consortium Mouse Build 39 (GRCm39) RefSeq assembly. Reads were aligned and counted in RStudio using the Bioconductor ‘RSubread’ (version 2.8.2) package [30]. Alignment was done with the ‘align’ function on the FASTQ files, creating sorted BAM files. ‘featureCounts’ function was used with the NCBI GRCm39 RefSeq Annotation and the commands: isGTFAnnotationFile = TRUE, countMultiMappingReads = FALSE, strandSpecific = 2, isPairedEnd = TRUE.

Differential gene expression (DGE) was done in RStudio using the Bioconductor ‘EdgeR’ [31–33] (version 3.36.0) package using a quasi-likelihood (QL) F test and GLM approach to do pairwise comparisons between groups. For the QL model, buffer type (TRIzol, PureLink Column, or PK buffer), organ type, and treatment (LPS vs. PBS) were used as factors. Differentially expressed genes were considered significant if the false discovery rate (FDR) was below 0.05. DGE figures were made with Bioconductor ‘EdgeR’, ‘Glimma’ [34] and ‘Vidger’ (https://bioconductor.org/packages/release/bioc/html/vidger.html) packages.

Principal component analysis was performed in RStudio with the Bioconductor ‘EdgeR’ package. The ‘plotMDS.DGEList’ function was used on the filtered and normalized EdgeR DGElist data with method="LogFC”, gene.selection="common”, and using the top 500 genes.

The overrepresentation of differenatially expressed genes (DEGs) within the reactome [35] pathways was performed with R package clusterProfiler (version 3.6) [36], separately for up- and down-regulated DEGs. A heatmap visualization was conducted with the ComplexHeatmap R package (version 2.3.1) [37], using pathways identified by both TRIzol and PK buffer conditions and with a ratio of at least 0.1 of differential vs. nondifferential genes present in a pathway. For clarity, only the top 2 pathways from each Reactome Level 1 category for each data point were taken into account. Pearson’s correlation coefficient for DEGs was calculated with R package Hmisc (https://CRAN.R-project.org/package=Hmisc).

Use of RSeQC requires .bam files created using the ‘samtools’ software kit so a separate set of .bam files was created using a pipeline of: ‘TrimGalore’ (https://www.bioinformatics.babraham.ac.uk/projects/trim_galore/, version 0.6.7) to trim adapters on the paired reads, ‘hisat2’ (version 2.2.1) [38] with “--rna-strandness RF” to align sequences to the mm10.ensGene.gtf assembly from UCSC (https://hgdownload.soe.ucsc.edu/goldenPath/mm10/bigZips/genes/) and samtools (version 1.13) [39] for generation of sorted and indexed .bam files.

## Results and discussion

### PIXUL-based RNA extraction

Proteinase K (named for its ability to hydrolyze keratin [40]) is a broad-spectrum serine protease which proteolytically inactivates nucleases even in the presence of sodium dodecyl sulfate (SDS) [14, 15, 40]. There is a long history of using this enzyme to isolate nucleic acids from biospecimens without the need to use hazardous solvents [12–15]. For example, we have before formulated proteinase K containing buffer to extract DNA [16, 17]. PIXUL can be used to isolate RNA with TRIzol [1]. As an alternative to TRIzol, we set out to develop a faster, lower-cost microplate PIXUL proteinase K-based protocol without the use of hazardous materials that can be done on the bench.

**Fig. 2:**
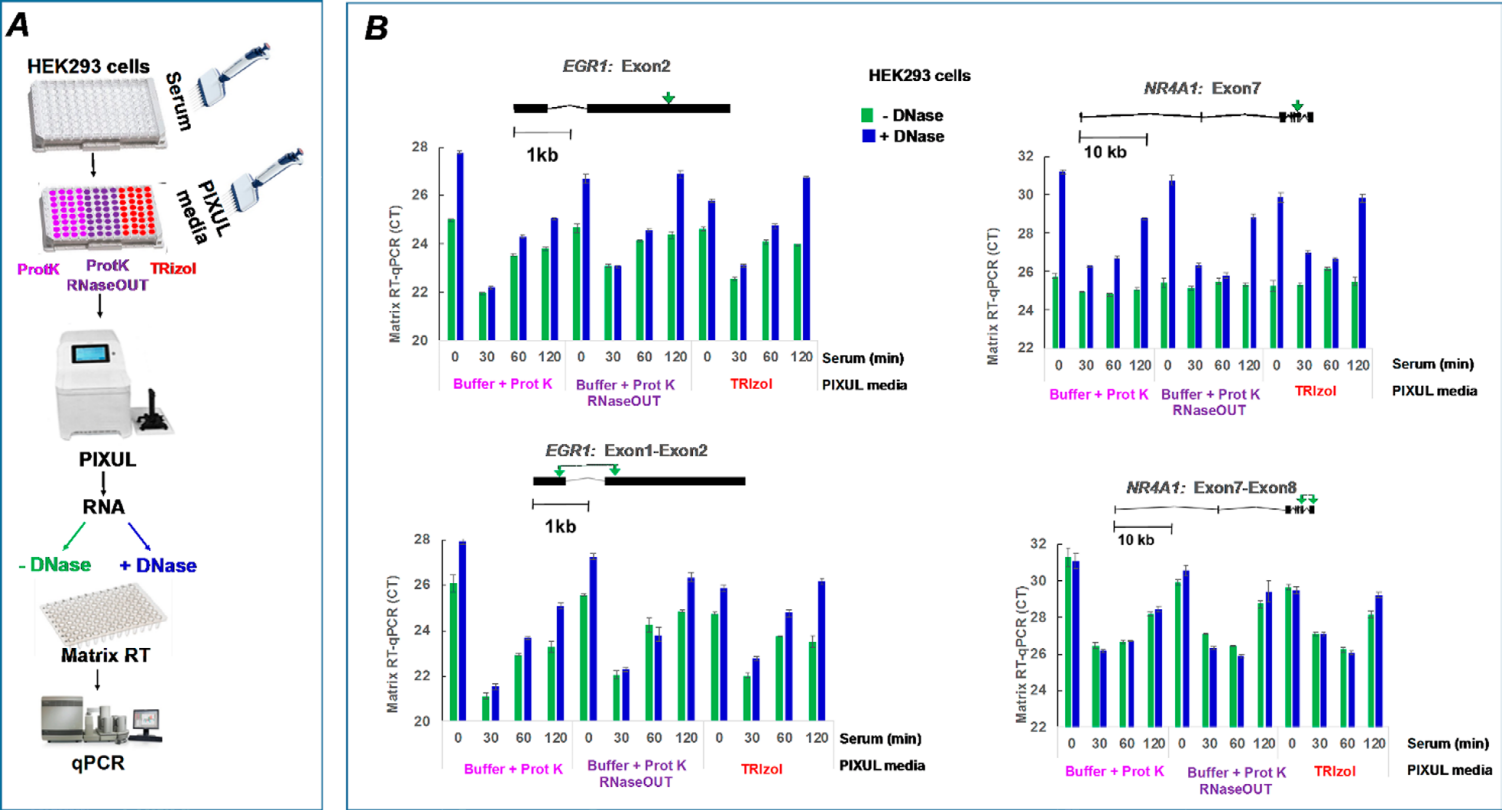
PIXUL-Matrix-RT-qPCR analysis with and without DNase of serum inducible genes in 96-well human HEK293 cultures. ***A***, Serum-deprived HEK293 96-well cultures were treated with serum for 0, 30, 60 and 120 min. Media was aspirated and replaced with either elution buffer-proteinase K, with and without RNaseOUT, or TRIzol. Plates were treated in PIXUL, and RNA was isolated and either treated (+ DNase) or not (-DNase) with DNase I. RNA was used in Matrix RT-qPCR with indicated primers. ***B***, Cartoons above graphs show primers used in qPCR. Data (mean ± SEM n = 4 qPCR) are expressed as cycle threshold (CT)

Serum-deprived human kidney HEK293 cells in 96-well plates are activated with serum treatment leading to a transient induction of immediate early genes such as *EGR1* [1]. PIXUL allows one to isolate analytes from 96-well plate cultures of HEK293 cells without sample transfer, greatly facilitating protocol development [1]. RNA isolated with PIXUL from serum-treated HEK293 cells showed the predicted transient increase in *EGR1* expression [1] and the increase was greater in DNase I treated samples with either the elution buffer-proteinase K or TRIzol method (Fig. S3). Figure 2 shows the qPCR data as cycle threshold, CT (lower CT values correspond to higher cDNA amount [41], CT lower by 1.0 is equal to 2-fold higher DNA levels), using primers that span (*EGR1*: Exon1-Exon2; *NR1A1*: Exon7-Exon8) or do not span (*EGR1*: Exon2; *NR1A1*: Exon7) exon regions. There was a marked transient serum-induced decrease in CTs for the *EGR1* and *NR4A1* primers spanning exons in the DNase I treated samples. In contrast, with the *NR4A1* Exon7 primers without DNase I treatment, both elution buffer-proteinase K and TRIzol samples had lower CTs (22 vs. 30–32) and failed to show serum induction, indicating gDNA contamination. The use of PCR primers that span exons does not completely overcome the misreading of the mRNA levels, particularly for the less abundant transcripts such as *NR4A1*, and underscores the fact that even with TRIzol DNase I treatment appears to be essential for preparing RNA used in RT-qPCR analysis. Using this protocol, the results suggest that RNaseOUT did not make a difference in extracting RNA from HEK293 cells.

**Fig. 3:**
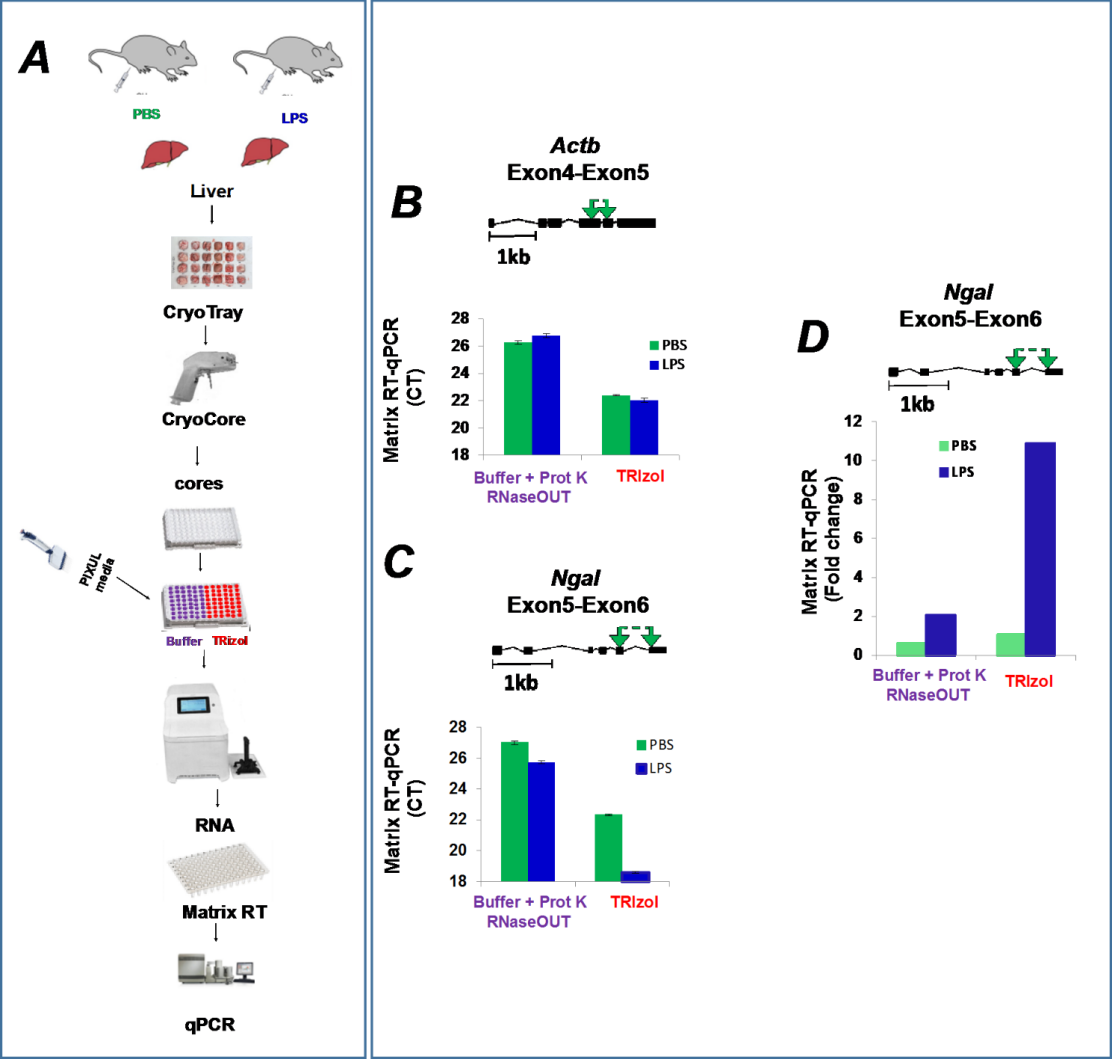
PIXUL-Matrix-RT-qPCR analysis of liver transcripts harvested from LPS, or PBS (control) treated mice and RNA isolated using either elution buffer-proteinase K or TRIzol from LPS. ***A***, Frozen mouse livers from LPS or PBS treated mice were sampled with CryoCore and jetted directly into wells of 96-well PIXUL plate. Samples were sonicated in either elution buffer-proteinase K or TRIzol. Isolated RNA was analyzed in Matrix-RT-qPCR. ***B-C***, Cartoons above the graphs show mouse primers spanning either *Actb* (B) or *Ngal* (C) exons used in qPCR. Data (mean ± SEM, n = 4 qPCR) are expressed as cycle threshold (CT). ***D***, *Ngal* mRNA expressed as fold change relative to *Actb* mRNA

Sepsis causes profound multiorgan changes in mRNA expression profiles, providing a system to test RNA isolation methods from organs using models of this syndrome [42–46]. Given its substantial RNase levels [47], liver could be a challenging organ to efficiently extract RNA from while minimizing degradation. Next, we tested the above protocol using either elution buffer-proteinase K or TRIzol RNA isolation from CryoCore samples taken from CryoTray-stored frozen livers of either LPS (endotoxin) or PBS (control) treated mice (Fig. 3A). Isolated RNA was used in Matrix RT-qPCR using exon-spanning primers to the housekeeping *Actb*, and multiorgan sepsis-inducible *Ngal* (*Lcn2*) genes [42, 45]. The CTs for *Actb* were much higher (lower mRNA levels) with the elution buffer-proteinase K method compared to TRIzol (Fig. 3B). Further, while there was LPS-induced substantial decrease in *Ngal* CTs in TRIzol-purified RNA, with the elution buffer-proteinase K method CTs were higher and there was only a small LPS-induced CT decrease (Fig. 3C). The striking LPS-response differences between the two methods is further underscored when the *Ngal* mRNA levels are normalized to *Actb* (Fig. 3D). These results suggest that the liver RNases degrade RNA even with the inclusion of RNaseOUT.

**Fig. 4:**
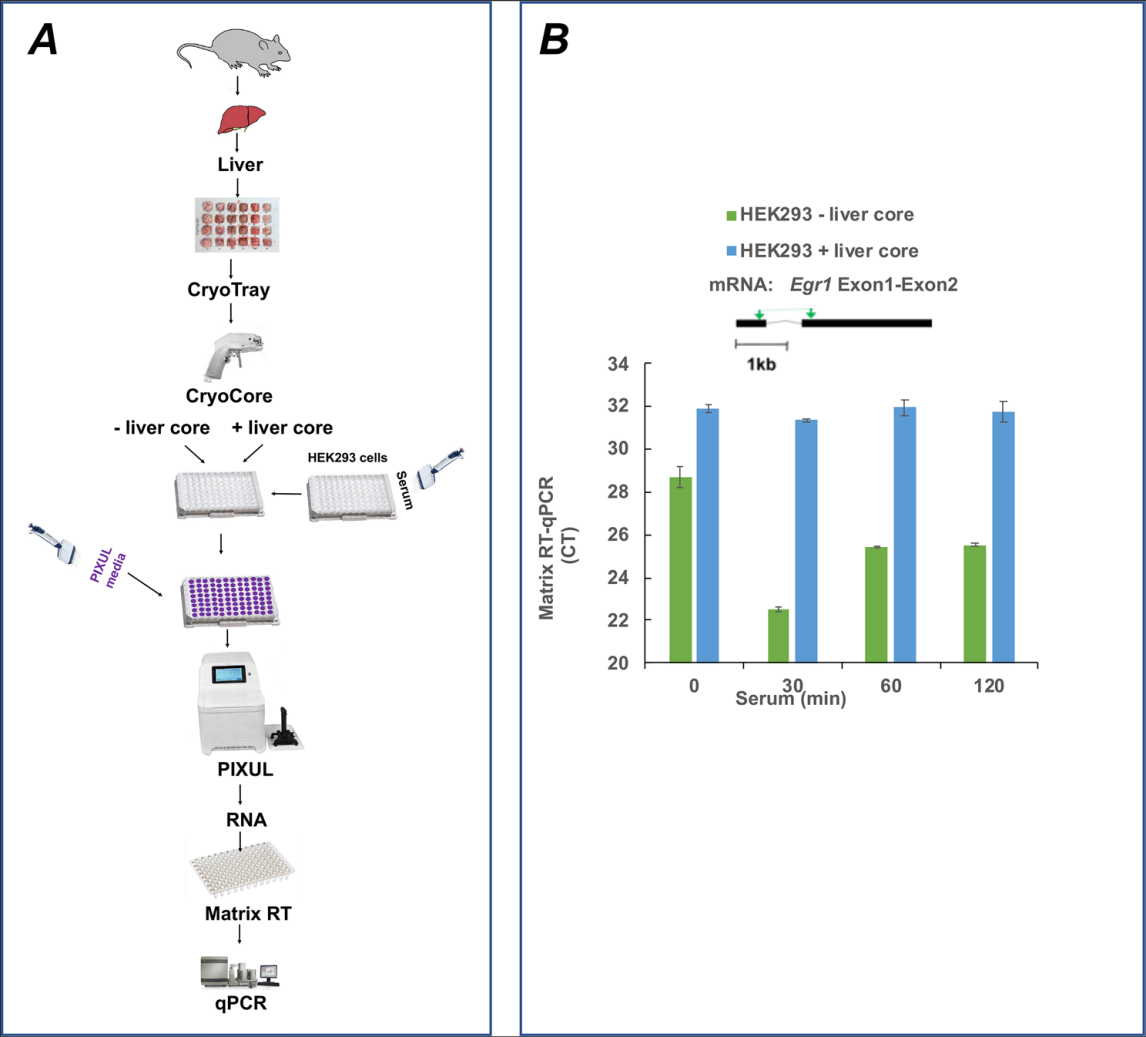
PIXUL-Matrix-RT-qPCR analysis of serum inducible *EGR1* gene in 96-well human HEK293 cultures co-sonicated with and without mouse liver cores. Serum-deprived HEK293 96-well cultures were treated with serum for 0, 30, 60 and 120 min. Media was aspirated and replaced with elution buffer-proteinase K. CryoCore liver samples were jetted or not to given wells, samples were sonicated in PIXUL, and RNA was isolated. RNA was used in Matrix RT-qPCR with indicated primers. Data (mean ± SEM, n = qPCR) is expressed as a cycle threshold (CT)

To further explore the role of RNases, CryoCore mouse liver samples were ejected into wells with elution buffer-proteinase K overlaying HEK293 cells from serum-treated cells. Plates were sonicated in PIXUL, RNA was isolated and *EGR1* mRNA levels were assessed in Matrix RT-qPCR using primers spanning Exon1-Exon2 region (Fig. 4A). Figure 4B shows that adding liver cores to the HEK293 cells increased CT values (less RNA) and no serum response was detected. These results provide evidence that the failure of the elution buffer-proteinase K, which works well in HEK293 culture (Fig. 2 and S3), to isolate RNA from livers might in part reflect RNase’s degrading activity in this organ.

**Fig. 5:**
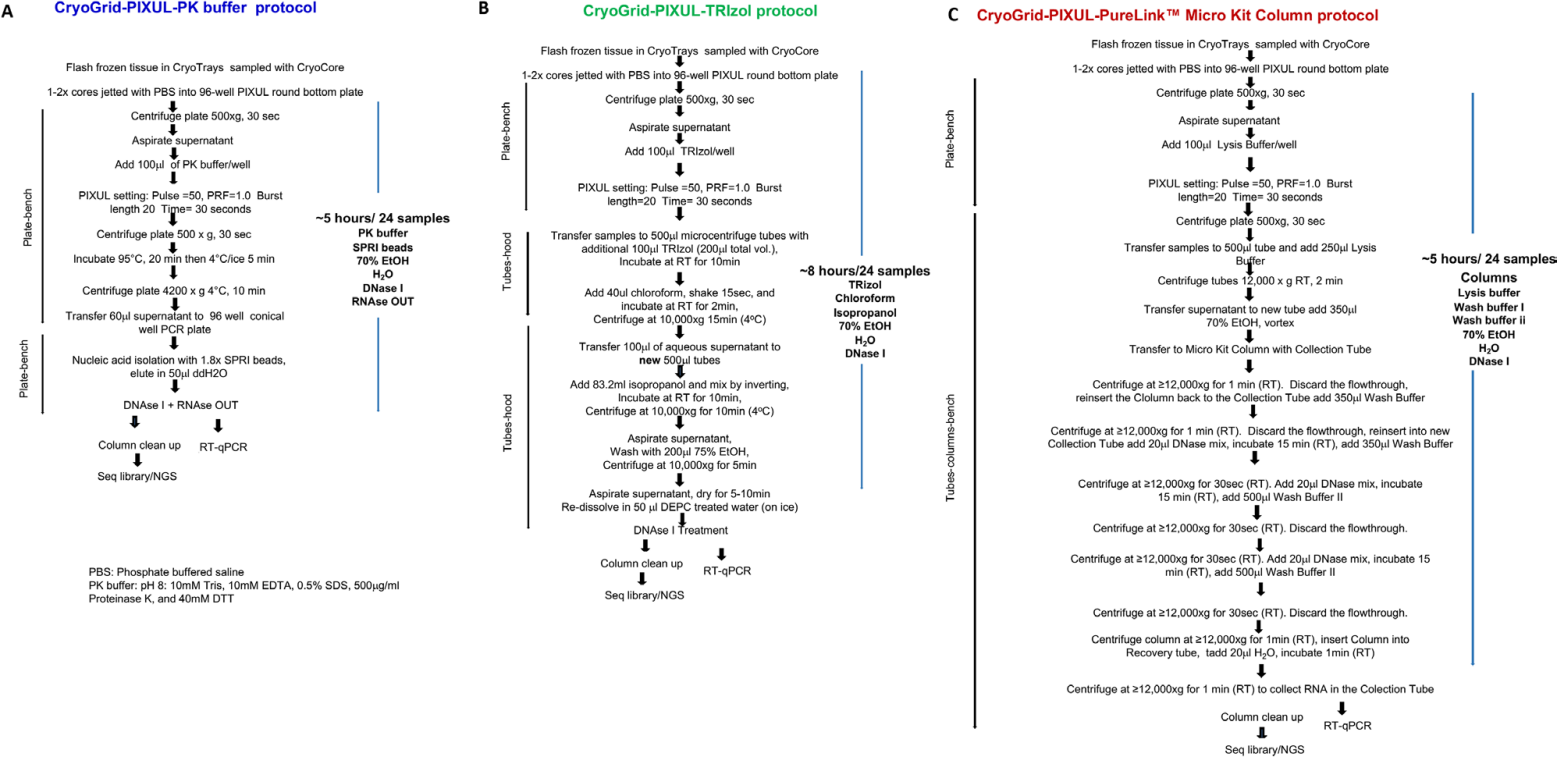
CryoGrid-PIXUL-RNA-qPCR/seq using PK buffer, TRIzol and PureLink column protocols for tissue RNA extraction. ***A***, The PK buffer protocol involves one transfer from PIXUL 96-well plate to a conical 96-well plate, does not use tubes or organic solvents and is done on an open bench. It requires SPRI beads, DNase I to remove gDNA and RNAseOUT to inhibit residual RNAses. This protocol takes approximately 5 h to isolate RNA from 24 tissue samples. ***B***, The TRIzol protocol involves transfer from PIXUL 96-well plate to tubes and then another transfer to a new set of tubes. Besides TRIzol this protocol uses two other organic solvents, chloroform and isopropanol, steps that need to be done under the hood. It requires DNase I treatment to remove gDNA. This protocol takes approximately 8 h to isolate RNA from 24 tissue samples. ***C***, The PureLink columnS protocol has many steps using columns and test tubes. It requires DNase I treatment. This protocol takes approximately 5 h to isolate RNA from 24 tissue samples

Using the above elution buffer-proteinase K recipe we unsuccessfully tested other known inhibitors of RNases including polyvinylsulfonic acid (PVS) [48] and bentonite [49] to isolate high-quality RNA from mouse livers. To increase the activity of proteinase K and decrease the activity of RNAses we tested adding chaotropic reagents including SDS and guanidium hydrochloride. We tested a range of pH and concentrations of EDTA, DTT and SDS to maximize tissue RNase inhibition and preserve RNA integrity from mouse livers using the PIXUL PK buffer protocols. SPRI are used to remove inhibitors. Given that SDS inhibits PCR and DNase activity, we added a SPRI beads step to isolate RNA and then treated the RNA prep with DNase I. Further, we added RNaseOUT to inhibit residual RNases during DNase treatment. The optimized PIXUL PK buffer along with TRIzol and PureLink protocols are shown for comparison in Fig. 5. Next the PK buffer protocol was compared to TRIzol and PureLink column methods to extract RNA from multiple organs from septic and control mice.

### Comparing PIXUL-PK buffer, PIXUL-TRIzol and PIXUL-PureLink column RNA isolation methods in multiple mouse organs

We used mouse organs from septic and control mice to compare RNA isolation using the PIXUL-PK buffer, PIXUL-TRIzol and PIXUL-PureLink protocols. Mouse brains, hearts, kidneys, and livers were harvested 12 h after either LPS (endotoxin) or PBS (control) intraperitoneal injection (IP) and frozen in CryoTrays. Organ cores (2 cores per well for brain and heart samples, 1 core per well for kidney and liver samples) were extracted with the CryoCore and jetted into three different 96-well plates for PIXUL treatment, and RNA was isolated using protocols as described and shown in Fig. 5. RNA yields and quality (absorbance 260/280 and 260/230) were estimated with the NanoDrop. With all three methods liver yielded the most RNA and heart the least. For all organs the RNA yields were highest with TRIzol and lowest using the PK buffer (Fig.S4A). Organs’ 260/280 (ranged 1.97–2.09) and 260/230 (ranged 1.80–2.35) absorbance ratios were nearly identical for the three methods (Fig. S4B). RIN, which is a qualitative – not quantitative – metric (Agilent Technical Support), is automatically derived using a proprietary algorithm originally developed for the Agilent Bioanalyzer. It assesses the entire electrophoretic trace of an RNA sample including the 18 and 28 S area ratios [50]. In the original Agilent study RIN was correlated with the quality of microarrays and RT-PCR data [50]. In contrast to that original publication, there are microarray studies where only 1% of probes were correlated with RIN [51]. Moreover, it has been shown that with selected library preparation protocols the number of mapped reads in RNA-seq demonstrated little or no correlation with RIN [52]. Our studies have shown that RIN was lower with the PK buffer protocol compared to either TRIzol or PureLink column for each organ as follows for PK buffer (PK), TRIzol (T) and PureLink column (PL): brain PK = 4.71 ± 0.15, TR = 5.83 ± 0.14 and PL = 6.0 ± 0.33; heart PK = 3.04 ± 0.15, TR = 5.64 ∓ 0.24 and PL = 5.9 ± 0.29; kidney PK = 2.84 ± 0.15, TR = 4.13 ± 0.15 and PL = 6.1 ± 0.13; and liver PK = 3.04 ± 0.13, TR = 4.73 ± 0.21 and PL = 5.1 ± 0.25 (p < 0.01 PK buffer compared to the other two methods for each organ) (Fig. S5A). It is possible that RIN was lower for PK buffer compared to the TRIzol and the PureLink columns protocols because it involves a heating step [51] and/or more fragmentation of RNA during PIXUL treatment. However, given that we used random hexamer priming, the PK buffer protocol has not reduced the quality of RT-qPCR transcript analysis compared to conventional TRIzol or columns (PureLink) based RNA isolation methods (below).

**Fig. 6:**
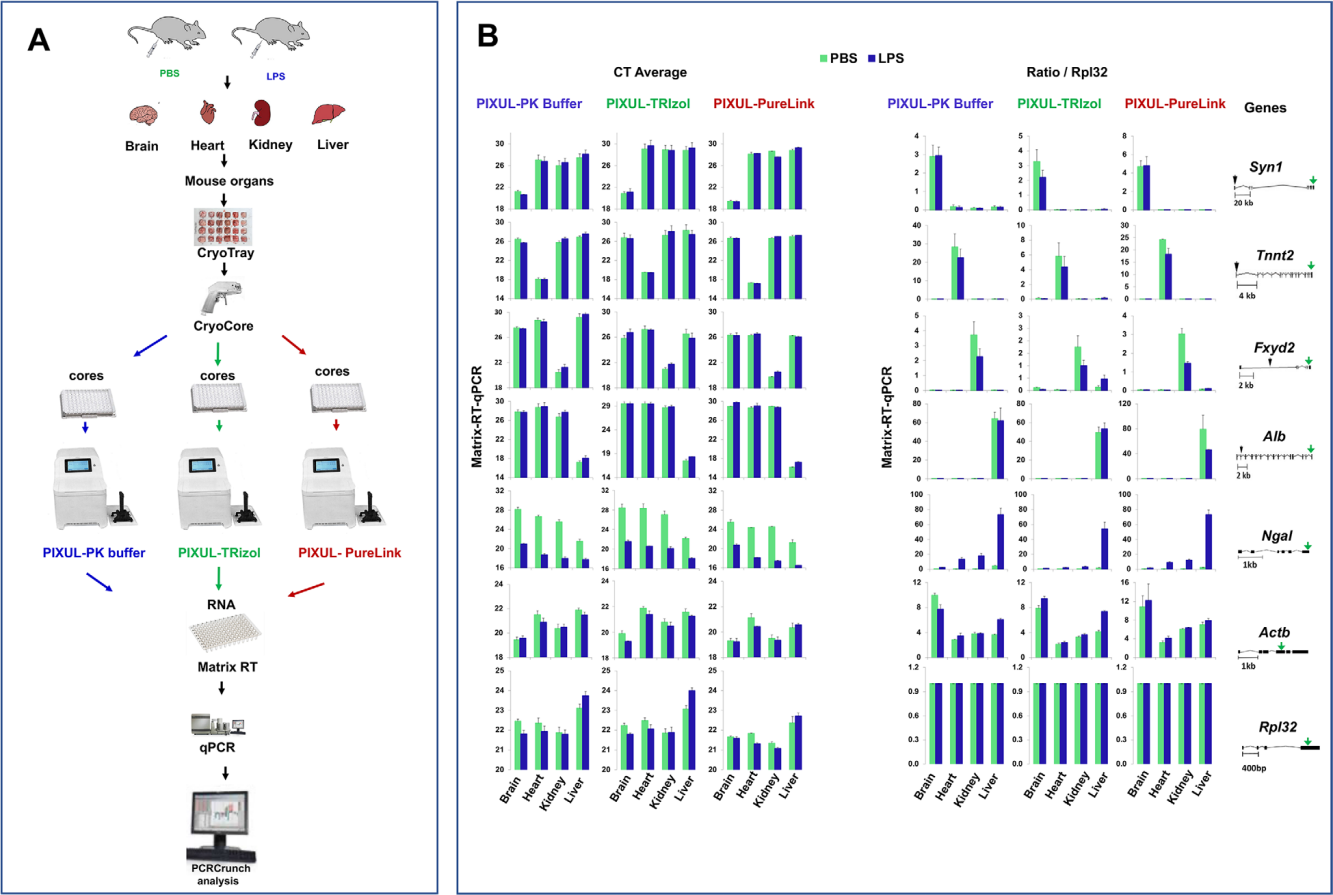
PIXUL-Matrix-RT-qPCR analysis of brain, heart, kidney and liver transcripts from LPS (endotoxin) or PBS (control) treated mice and RNA isolated using either SDS buffer-proteinase K (PK buffer), TRIzol or PureLink column. ***A***, Frozen mouse brains, hearts, kidneys, and livers from LPS or PBS treated mice in CryoGrid were sampled with CryoCore, and then cores jetted directly into wells of 96-well PIXUL plate. Samples were sonicated in either SDS buffer-proteinase K (PK buffer), TRIzol or PureLink lysis buffer. Isolated RNA was analyzed in Matrix-RT-qPCR. ***B***, Data (mean ± SEM, n = 2 sets of each of frozen organ) are either expressed as CTs (*left*) or normalized to *Rpl32* (*right*) housekeeping gene. Cartoons of mouse genes are shown on the right (the green arrow shows the location of the PCR primer)

Matrix-RT-qPCR was used next to compare the PK buffer, TRIzol, and PureLink column protocols (Fig. 6A) to assess the well-described organ-specific, sepsis-induced, and housekeeping mRNA expression patterns [1, 42, 45]. Data are shown as both CTs and as a ratio to the *Rpl32* housekeeping mRNA (Fig. 6B). *Syn1, Tnnt2, Fxyd2, Alb* show striking organ-specific signals for brain, heart, kidney and liver respectively. The housekeeping genes (*Actb* and *Rpl32*) did not show significant organ specificity. For organ-specific genes there was a moderate LPS-induced downregulation in the heart (*Tnnt2*) and kidney (*Fxyd2*). As illustrated by CTs, constitutive *Ngal* expression is much higher in the liver compared to the other organs. *Ngal (Lcn2)* expression was induced by LPS treatment in all four organs as illustrated by decreased CT and increased ratio to *Rpl32*. Elevated LPS endotoxin-induced *Ngal/Lcn2* expression in liver as well as inducible expression across multiple organs has also been shown in the mouse cecal ligation and puncture (CLP) sepsis model [42]. The results obtained with the PIXUL-PK buffer were indistinguishable from those with either the PIXUL-TRIzol or PureLink methods, suggesting that the three methods are equivalent when measuring expression of these genes by RT-qPCR (Fig. 6).

### RNA-seq analysis of mouse organs

**Fig. 7:**
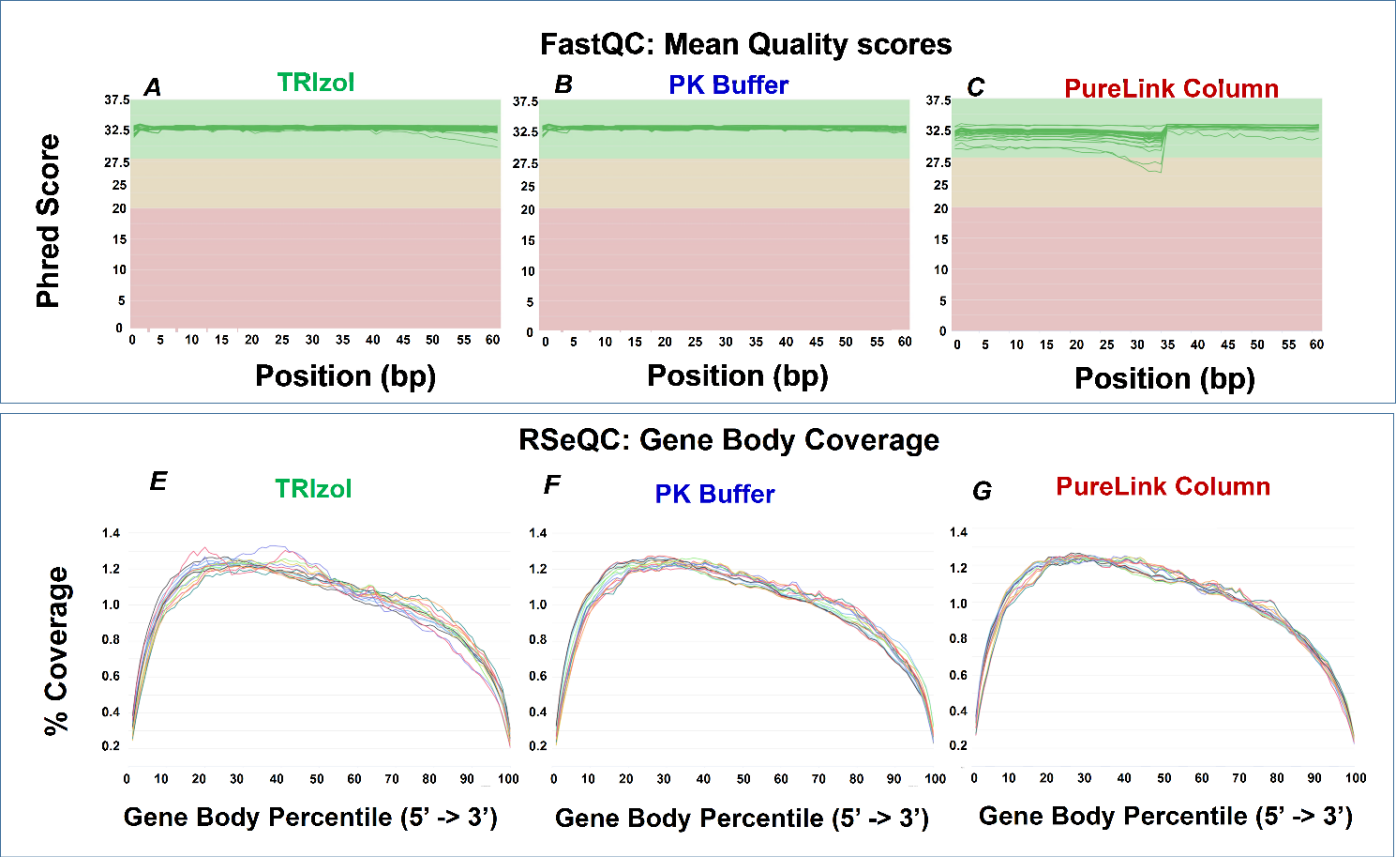
Sequencing quality. ***A-C***, FastQC ‘Per Base Sequence Quality’ scoring of RNA-seq for TRIzol (A) isolated samples, PK buffer (B) and PureLink Column (C) isolated samples. Each chart showing Phred score on the Y-axis and base pair position on the X-axis. ***E-G***, RSeqQC ‘Gene Body Coverage’ of read coverage percentage of TRIzol (E), PK Buffer (F) and PureLink Column isolated samples along the length of gene bodies (X axis, *Gene Body Percentile*)

**Fig. 8:**
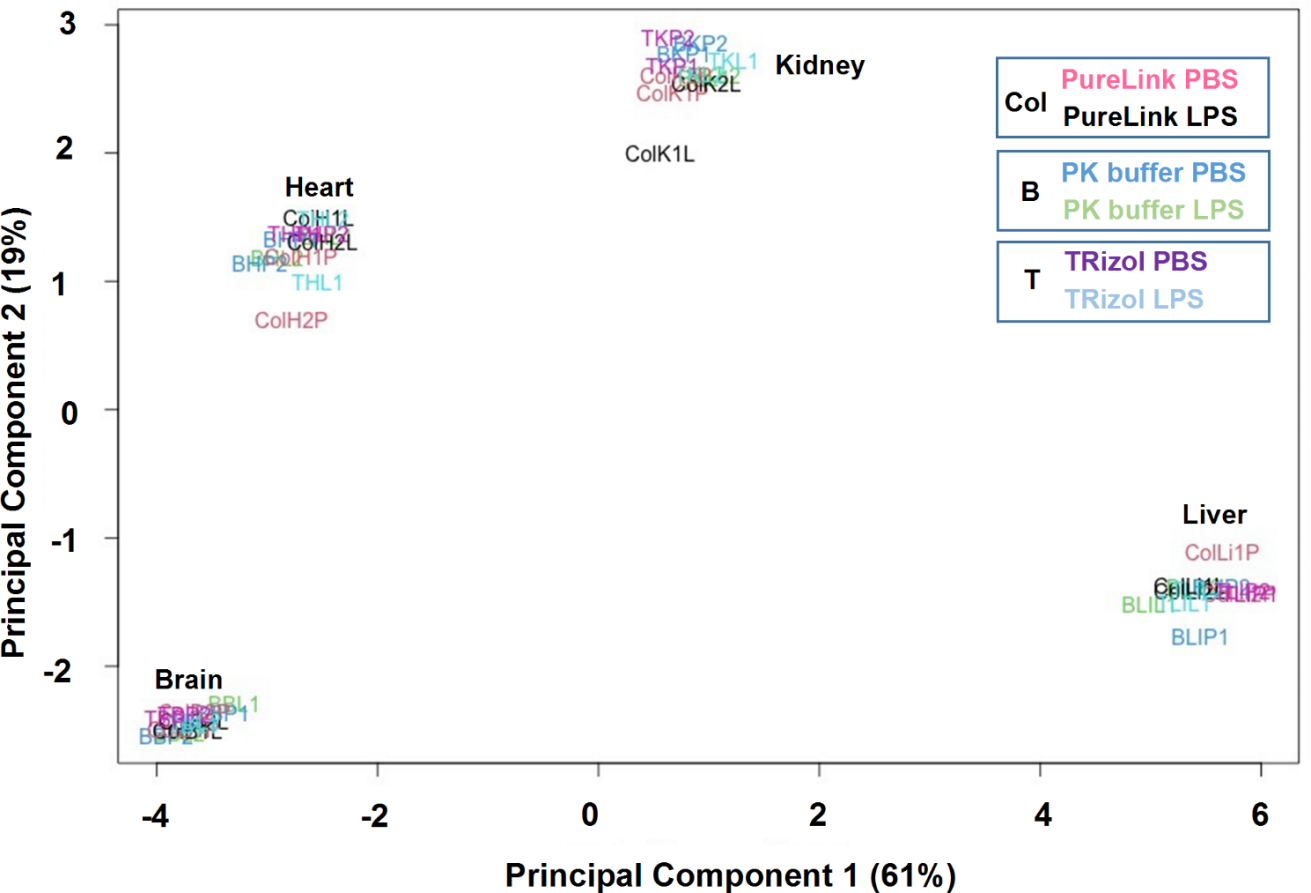
Principal component analysis (PCA) of brain, heart, kidney and liver RNA-seq using RNA isolated with the PIXUL-PK buffer, PIXUL-TRIzol and PIXUL-PureLink column methods from LPS and PBS IP injected mice. Data show two replicates for each organ for LPS and PBS IP-treated mice

**Fig. 9:**
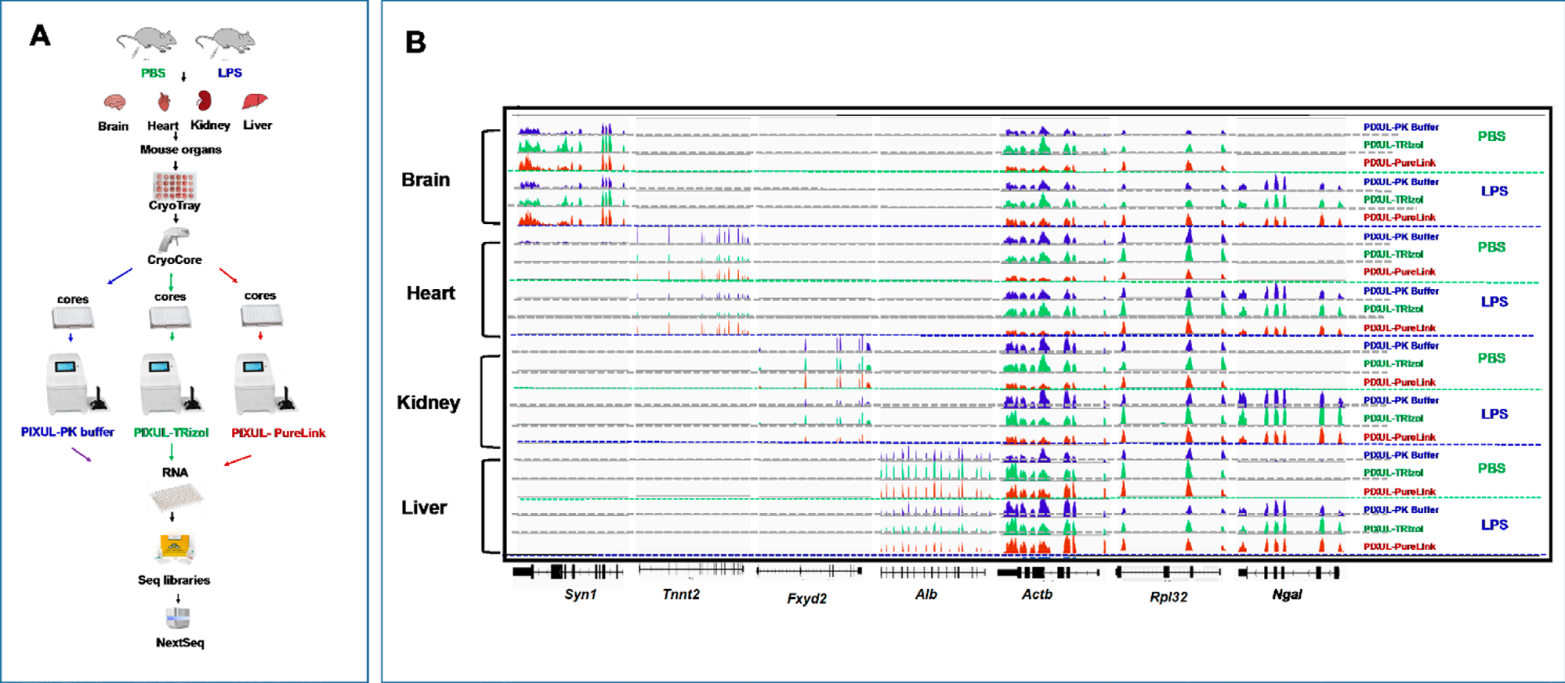
CryoGrid-PIXUL-RNA-seq analysis of brain, heart, kidney and liver transcripts from LPS or PBS treated mice and isolated using either PK buffer, TRIzol or PureLink column. ***A***, Mouse brains, hearts, kidneys and livers from LPS or PBS treated mice were frozen in CryoGrid, CryoCore sampled and then jetted directly into wells of 96-well PIXUL plate. Samples were sonicated in either PK buffer, TRIzol or PureLink lysis buffer. Sequencing libraries were generated using Zymo-seq RiboFree kit and sequenced on NextSeq2000 employing a dual-index, paired-end, 61 base read length (PE61). ***B***, IGV mRNA tracts for organs specific at 3’-ends of organ-specific (brain: *Syn1*, heart: *Tnnt2*, kidney: *Fxyd2* and liver: *Alb)*, house-keeping *(Actb*), sepsis-inducible (*Ngal(Lcn2*)) and sepsis-down-regulated (*Tek*) genes. Data shown represent one of two RNA-seq done on two different sets of each frozen organ

RNA-seq provides a transcriptome-wide view at gene expression which we used next to compare the three tissue RNA extraction protocols. Zymo-Seq RiboFree Kit was used to prepare libraries which were then sequenced on Illumina NextSeq2000 (Fig. 7). All samples for both TRIzol and PK buffer methods of RNA isolation had mean quality per base Phred scores greater than 30 across all base pairs, equating to greater than 99.9% accuracy in all base calls, whereas PureLink samples were generally above 30 and had a low Phred score of 25.5 for the heart samples (Fig. 7A-C) [29]. RSeqQ gene body read coverage analysis [27] of RNA isolated with PureLink, TRIzol, or PK buffer showed even distribution across the gene bodies (Fig. 7D-F) suggesting that coverage of transcripts from all three isolation methods is both uniform and nearly indistinguishable. Transcript integrity number (TIN) is a metric that assesses RNA integrity using RNA-seq data [28]. Fig.S5B illustrates that each organ’s TIN is similar with no correlation to the RIN values, indicating that by this metric the RNA integrity is the same for the three RNA isolation methods. Scatter plot analysis of RNA-seq normalized count data showed excellent agreement between PK buffer and TRIzol RNA isolation methods (R = 0.984–0.991), between PK buffer and PureLink column methods (R = 0.983–0.992), and between TRIzol and PureLink column methods (R = 0.970–0.988) (Fig.S6A-C). Principal component analysis (PCA) of RNA-seq data showed clear organ-specific gene clusters, and data from all three isolation methods cluster together (Fig. 8). The IGV browser RNA-seq screenshots along the organ-specific *Syn1, Tnnt2, Fxyd2, Alb*, the house-keeping, *Actb* and the LPS-responsive *Ngal* genes recapitulate the qPCR results (Figs. 6B and 9 A-B).

**Fig. 10:**
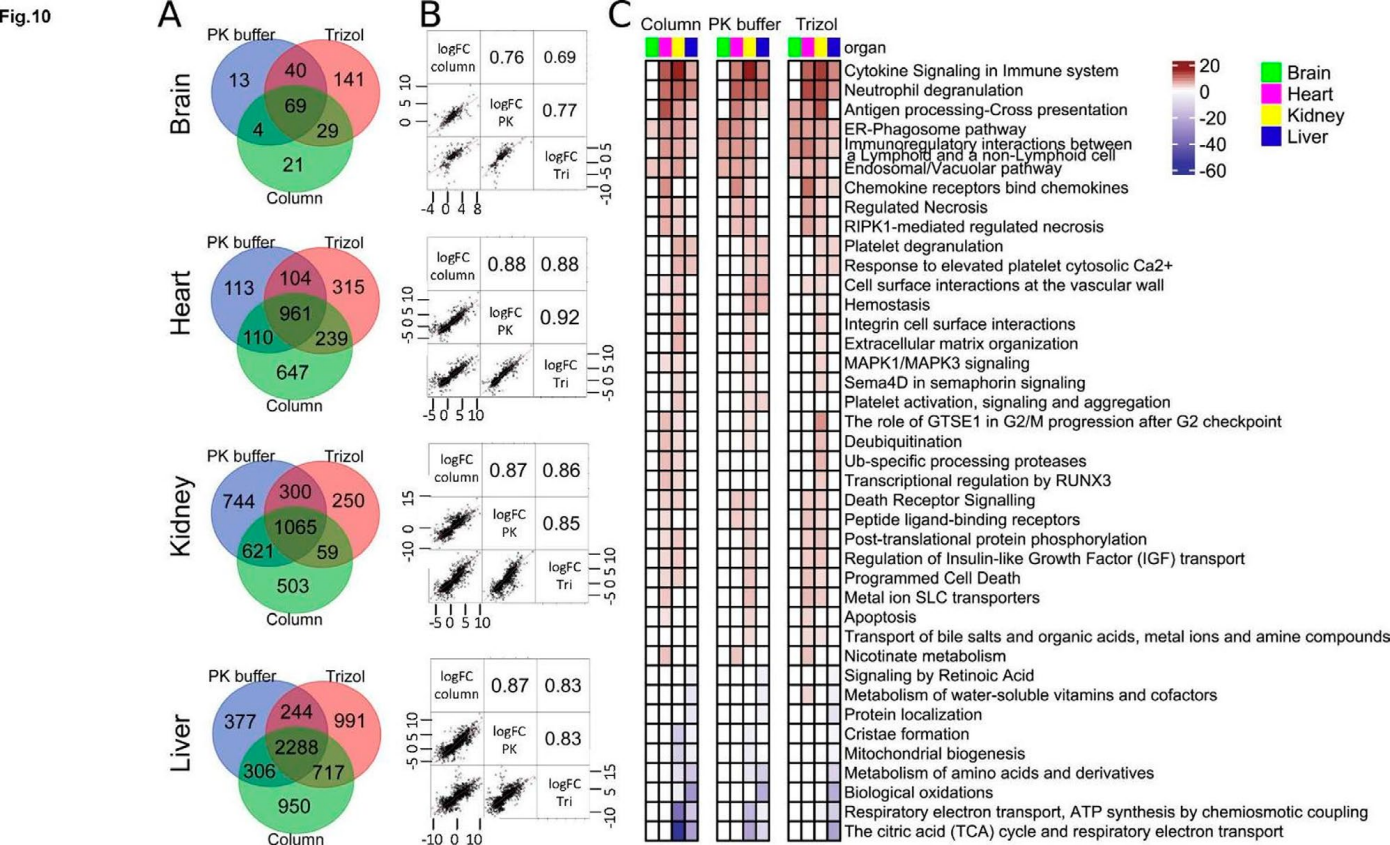
Differential gene expression and Pathway analysis. ***A***, Venn diagrams depicting the overlapping numbers of up-and down-regulated DEGs (adj. p-value < 0.05) for mouse organs following LPS endotoxin challenge between the PK buffer, TRIzol and PureLink column RNA isolation methods. ***B***, Organ’s Pearson’s correlation coefficient for DEGs comparing PK buffer (PK), TRIzol (Tri) and PureLink columns (column) RNA isolation methods with each other. ***C***, Significantly altered Reactome pathways (adj. p-value < 0.05) across the organs following LPS endotoxin IP injection for the PureLink columns, TRIzol and PK buffer RNA isolation methods. The Reactome pathways with a differential gene ratio higher than 10% are shown. The p-value is on the PHRED scale and is reversed for down-regulated pathways

We carried out differential gene expression analysis with ‘EdgeR’ as another way to compare the three RNA isolation methods. Mean difference (MD) plots showing pairwise comparisons between the three isolation methods show very few statistically significant differences in differentially expressed transcripts between the three methods (Fig.S7A-C). In contrast to this comparison, MD plots of transcripts isolated with each method illustrate large numbers of statistically significant LPS-induced or repressed genes (LPS vs. PBS comparisons) in the heart, kidney, and the liver, although there were many fewer LPS-responsive genes in the brain (Fig.S8). Numerical values are shown in Fig. S9. With all three methods, after filtering lowly-expressed genes, there were more than 21,000 transcripts detected in each one of the four organs (Fig.S9A-D). The number of transcripts for each organ differentially expressed between each of the pairwise comparisons of RNA preps were as follows. TRIzol and PK buffer were the most similar with TRIzol detecting at most 20 (heart) genes not detected in the PK buffer and PK buffer detecting at most 16 (heart) genes not detected in the TRIzol method (Fig.S9A). The comparison of PK buffer and PureLink column was second most similar with PureLink detecting at most 133 (brain) genes not detected with PK buffer and PK buffer detecting at most 69 (brain) genes not detected with the PureLink method (Fig.S9B). PureLink columns and TRIzol showed the most differences between methods with PureLink detecting at most 211 (heart) genes not detected with TRIzol and TRIzol detecting at most 221 (heart) genes not detected with the PureLink method (Fig.S9C). The number of LPS-(upregulated//downregulated) transcripts for each of the isolation methods were as follows: brain- PK 158//15, TR 328//81, PL 107//16; heart- PK 1086//749, TR 1067//1194, PL 1018//939; kidney- PK 1726//1944, TR 1256//1009, PL 1125//1123; liver- PK 2241//1884, TR 2657//2559, PL 2124//2137 (Fig.S9D). Thus, the percent of genes upregulated//downregulated in response to LPS was as low as < 1%//1.5% in the brain but as high as 11%//12% in the liver. The low endotoxin response in the brain is not unexpected given the blood-brain barrier. In each organ there were LPS-induced DEGs seen using one method but not the others and vice versa. But in each case (except for the brain) there were substantially more LPS DEGs that were shared by the three methods than not (Fig. 10A). The mean organ’s Pearson’s correlation coefficient for DEGs comparing the three RNA isolation methods with each other were similar and ranged as follows: brain 0.69–0.77; heart 0.88–0.92, kidney 0.85–0.87 and liver 0.83–0.87 (Fig. 10B).

Reactome PK buffer, TRIzol, and PureLink RNA-seq data analysis of LPS-induced DEGs for each organ identified nearly identical pathways well described in models of sepsis [42, 44, 53]. This included such pathways as cytokine signaling in the immune system, neutrophil degranulation, signaling by interleukins, platelets degranulation and several others (Fig. 10C). Altogether these analyses using RNA-seq data demonstrate that the three CryoGrid-PIXUL based methods of RNA extractions yield similar results.

**Limitations.** The mouse heart, the smallest organ (< 150 mg) used in this study, can be sampled several times with the CryoCore (Fig.S2). Still, there are situations where the available tissue fragments are smaller than the mouse heart (e,g, clinical samples). Here, to be effective, miniature trephine gauges will be needed for multisampling very small tissue fragments in research and clinical settings (e.g. for genomics, epigenetics, transcriptomics, proteomics and histology). The current CryoTrays hold 24 tissue fragments. For small tissue samples, CryoTrays with 48 or 96-wells would further save cryostorage space.

Human operators are prone to error [54]. Although using the iPad display largely mitigates human error using the 24-well CryoTray, with 48 or 96 samples on the CryoTray the introduction of computer vision and/or automation would provide an error-free sampling method. In this regard, we suggest that the application of the CryoGrid-PIXUL-Matrix-RNA-qPCR system to assays of organ-specific genes (Fig. 6) provides a convenient way to develop and test computer vision technology-assisted high throughout tissue sampling [55, 56].

For convenience (mobile iPad and iPhone) we used easy to-use no-cost basic Google Drive data storage. There are situations where high level data protection is required (e.g. human biopsies) and which might vary by institutions and/or labs. The integrated CryoGrid-PIXUL-Matrix system with the QR coded CryoTrays should be easily adaptable to any cloud storage system and meet stringent data protection requirements for secure data storage protection.

Although slow and taxing when using multiple samples, TRIzol is considered the “gold standard” method for RNA isolation [11] which, along with established PureLink columns, we used as the benchmarks in this study. The RIN was higher with TRIzol and PureLink compared to PK buffer (Fig.S5B). Nonetheless, TIN [28], RNA-qPCR and RNA-seq results obtained with the PK buffer were similar to TRIzol and PureLink column (Figs. 6 and 7 and S5-S9). But the PK buffer method has the advantage of being faster, biosafe, fewer steps, less labor-intensive and – given that it uses microplates – more suitable for automation. Still, there could be RNA species (e.g. small RNAs) where TRIzol and/or columns perform better than the PK buffer protocol, or vice versa. More detailed comparative analysis is needed to find putative RNA species preferentially lost in one protocol versus the other protocol.

## Conclusions

After protein and DNA, RNA is the most studied biomolecule (PubMed, Google Scholar). RNA is increasingly being used as a clinical diagnostic analyte [57]. Understanding RNA biology in tissues is critical in health and disease as well as therapeutic interventions (e.g., small molecules [58] and the rapidly emerging field of epigenome editing [59]). Freezing of tissues is widely used in research and clinical settings because it provides a practical way to preserve biospecimens for analysis. And yet, sampling of frozen tissues remains tedious. We developed a user-friendly cryostorage method and a hand-held sampling tool to interrogate tissues, CryoGrid system (Fig. 1, S1-S2), which we integrated with PIXUL sonicator for high throughput tissue RNA extraction.

Advantages of CryoGrid-PIXUL over existing tissue processing platforms are as follows.

i. It is a user-friendly method to freeze, track and sample tissues.
ii. One CryoCore 1–2 mm^3^ (1-2 mg) tissue sample is sufficient for RNA-seq, thus allowing the same frozen tissues (e.g. as small as mouse heart) to be sampled multiple times for other analyses as well as histology.
iii. After sampling, and unlike commercially available RNA extraction platforms, the separate initial tissue homogenization step is not needed.
iv. Virtually every disease and therapeutic agents’ effects are systemic conditions. The CryoGrid-PIXUL platform is well suited for parallel processing of multiple organs from model systems and for testing multi-organ effects in pre-clinical studies [46, 59, 60].
v. Ability to process dozens of samples at the same time mitigates batch effects [61].
vi. Transcriptomic, epigenetic, and proteomic analyte preparation can be done in parallel on the same PIXUL plate.
vii. The CryoGrid system could be integrated with a variety of sonicators.
viii. Using the CryoGrid-PIXUL system will expand the role of RNA-seq in pre-clinical studies and clinical settings [57].

Advantages of PIXUL-PK buffer over existing protocols are as follows.

i. The PK buffer method provides a way to isolate RNA without the use of hazardous solvents and as such can be done on the bench – a safety hood is not needed.
ii. Unlike TRIzol or column protocols, which use tubes, the PK buffer RNA extraction procedure is done in 96-well plates, making it adaptable for automation.
iii. Has the potential to be used for tissue RNA extraction for single-cell (scRNA)-seq.
iv. Cells or organoids grown in 96-well plates can be used directly to extract RNA in PIXUL without sample transfer [1].

### Electronic supplementary material

Below is the link to the electronic supplementary material.

Supplementary Material 1

## Data Availability

Sequence data was deposited in Gene Expression Omibus database under entry GSE199598.
