# Supplementary material for: CryoGrid-PIXUL-RNA: high throughput RNA isolation platform for tissue transcript analysis
Source: BMC Genomics. 2023 Aug 8;24:446. doi: 10.1186/s12864-023-09527-7 (PMC10408117; doi:10.1186/s12864-023-09527-7)
Supplement: Supplementary file 1 — Supplementary Material 1 [file 12864_2023_9527_MOESM1_ESM.pdf]

SUPPLEMENT INFORMATION

CryoGrid-PIXUL-RNA: High throughput RNA isolation platform for tissue transcript analysis

Scott A. Schactler[1,2], Stephen J. Scheuerman[1,2], Andrea Lius[1,2], William A. Altemeier[1,3], Dowon An[1,3], Thomas J. Matula[4,7], Michal Mikula[5], Maria Kulecka[5,6] , Oleg Denisenko[1], Daniel Mar[1,2] and Karol Bomsztyk[1,2,7*]

[1]UW Medicine South Lake Union, University of Washington, Seattle, WA 98109, USA, [2]Institute for Stem Cell and Regenerative Medicine, University of Washington, Seattle, WA 98109, USA, [3]Center for Lung Biology, University of Washington, Seattle, WA 98109, USA, [4]Center for Industrial and Medical Ultrasound, Applied Physics Laboratory, University of Washington, Seattle, WA 98195, USA, [5]Department of Genetics, Maria Sklodowska-Curie National Research Institute of Oncology, 02-781 Warsaw, Poland, [6]Department of Gastroenterology, Hepatology and Clinical Oncology, Centre for Postgraduate Medical Education, 01-813 Warsaw, Poland and [7]Matchstick Technologies, Inc, Kirkland, 98033 WA, USA.

*Address for correspondence:
Karol Bomsztyk, MD
UW Medicine at SLU
Box 358050
University of Washington,
Seattle, WA 98109 USA
karolb@uw.edu
206-616-7949

FIGURES

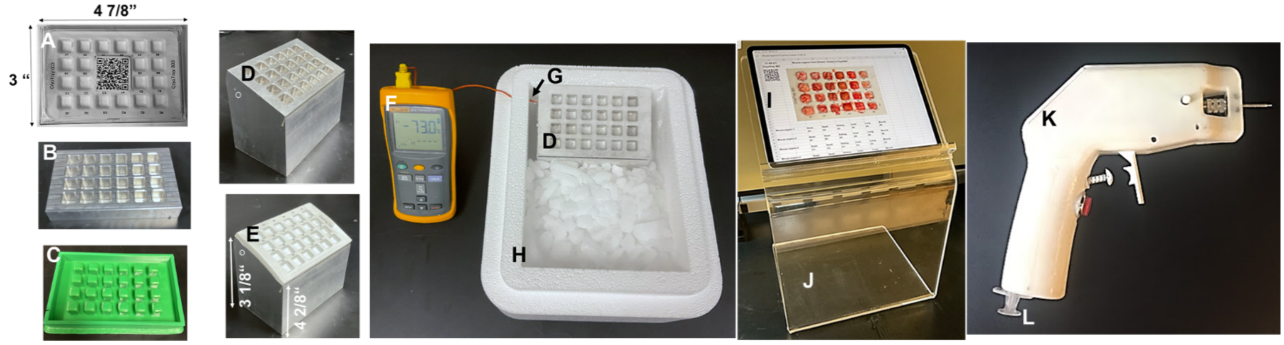

Fig.S1. CryoGrid system components. CryoTray dimensions of a standard plate (*A*) are casted in-house by placing polystyrene sheets (0.4mm) on heated (135°C) 24-cuboid sockets aluminum block (*B*) and then overlaying and pressing the sheet with a matching 24-cuboid plugs plastic stamp (*C*). The CryoTray dimensions match an off-the-shelf lid (Cytiva #7704-1001). QR code sticker is secured on the inside of the lid and linked to Google Drive applications. CryoBlock is a 24-pockets aluminum block with a tilted top (30°) to improve ergonomics (*D*) which serves as a CryoTray receptacle (*E*) providing access for coring tissues. Fluke thermometer (*F*) with a thermocouple probe (*G*) is inserted into the CryoBlock (*D*) and monitors temperature (<-70°C). CryoBlock is chilled in an off-the-shelf 11"x11"x9" Styrofoam box (CryoBox) containing dry ice (*H*). iPad (*I*) on a stand (*J*) displays a layout of tissues and metadata from an online Google sheet by scanning CryoTray QR code to guide tissue sampling with the CryoCore (*K*). CryoCore is a miniature coring drill with a trephine burr. Trephine is made from stainless steel tubing shaft (2mm x 30mm) and a smaller diameter tip (1mm x 1-2mm) with axially oriented cutting teeth around the tip. A push-button switch (*red*) activates the motor to drill into the tissue and then extract a core. Trephine has two holes with axes oriented perpendicular to shaft. A clamping mechanism uses two segments of silicone tubing with semicircular notches at their ends to close around the shaft holes, creating a seal. The clamp is actuated by a cable in a manner similar to a bicycle caliper brake. A pump mechanism utilizes a plunger and cylinder and two check valves to both draw buffer from a 5ml syringe (*L*) and to generate a fluid jet to eject the extracted core (18,62).

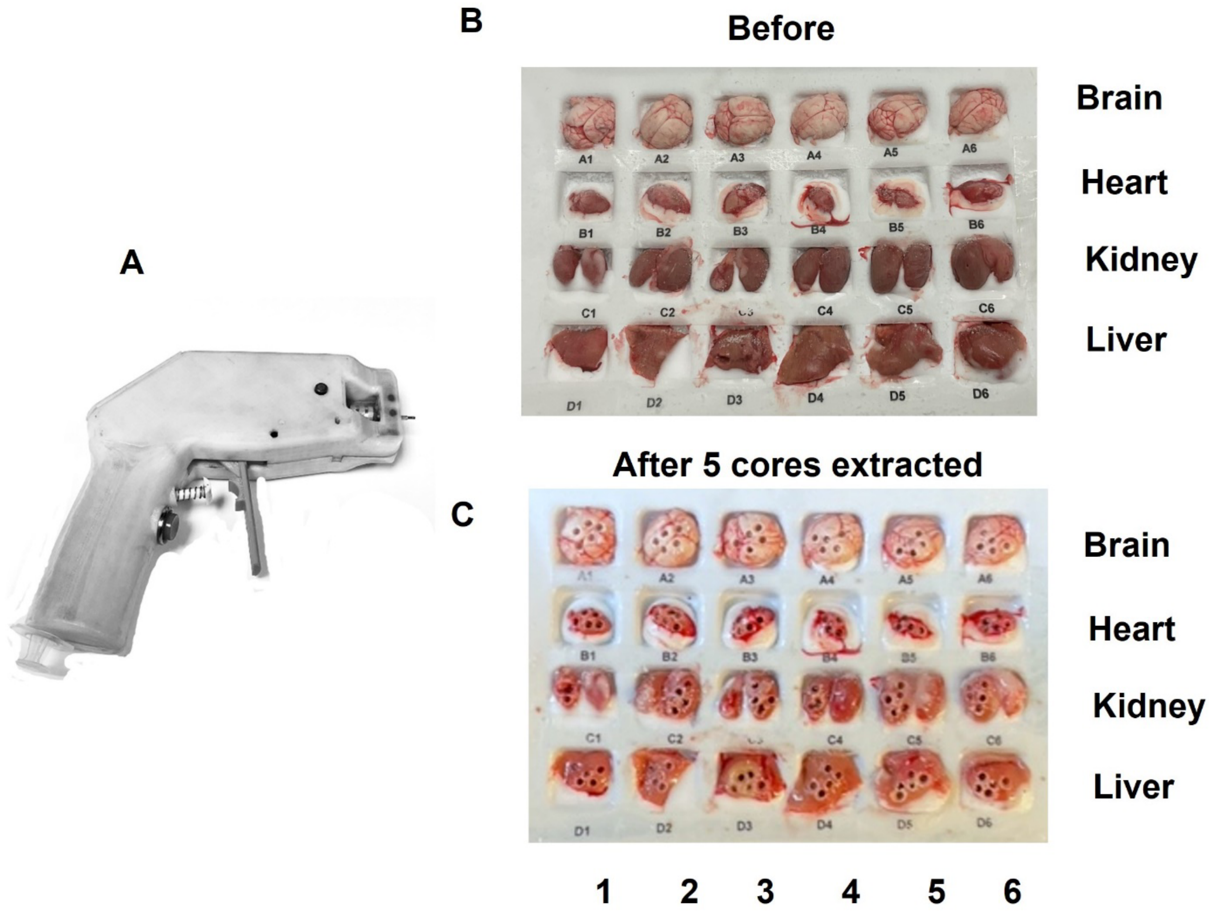

Fig.S2. CryoTray with tissues before and after multiple CryoCore sampling. ***A***, CryoCore was used to sample mouse organs. ***B-C***, CryoTray with frozen mouse organs were photographed before and after multiple CryoCore sampling, illustrating the size (~1-2 mm^3) and the number of cores that can be extracted from an organ as small as the mouse heart (<150mg).

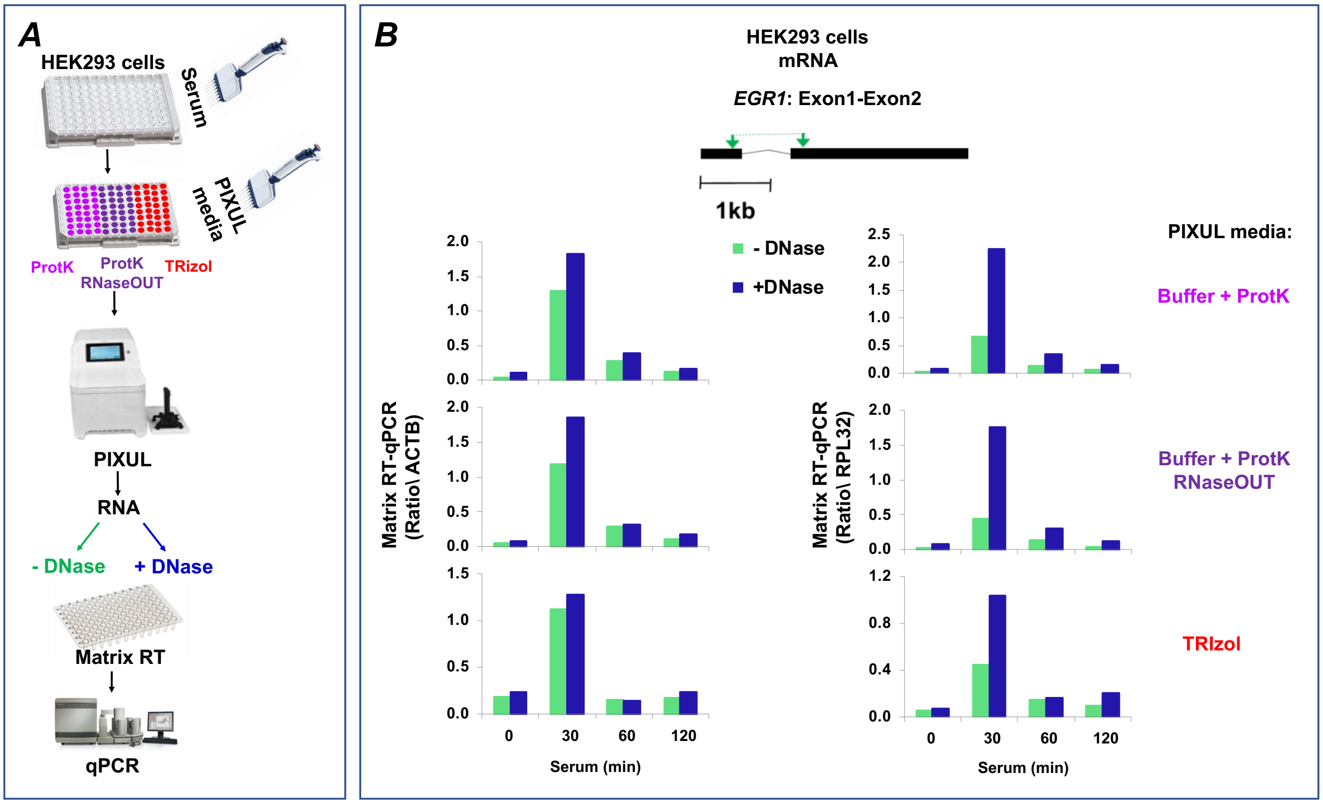

Fig.S3. PIXUL-Matrix-RT-qPCR analysis with and without DNase of serum inducible *EGR1* in 96-well human HEK293 cultures. ***A,*** Serum-deprived HEK293 96-well cultures were treated with serum for 0, 30, 60 and 120min. Culture media was aspirated and replaced with either elution buffer-proteinase K, with and without RNaseOUT, or TRIzol. Plates were treated in PIXUL, and RNA was isolated and either treated (+DNase) or not (-DNase) with DNase I. RNA was used in Matrix RT-qPCR. ***B,*** Human *EGR1* primer spanning Exon1-Exon2 used is shown. *EGR1* mRNA data are expressed as a ratio to *ACTB* (*left*) and *RPL32* (*right*) genes.

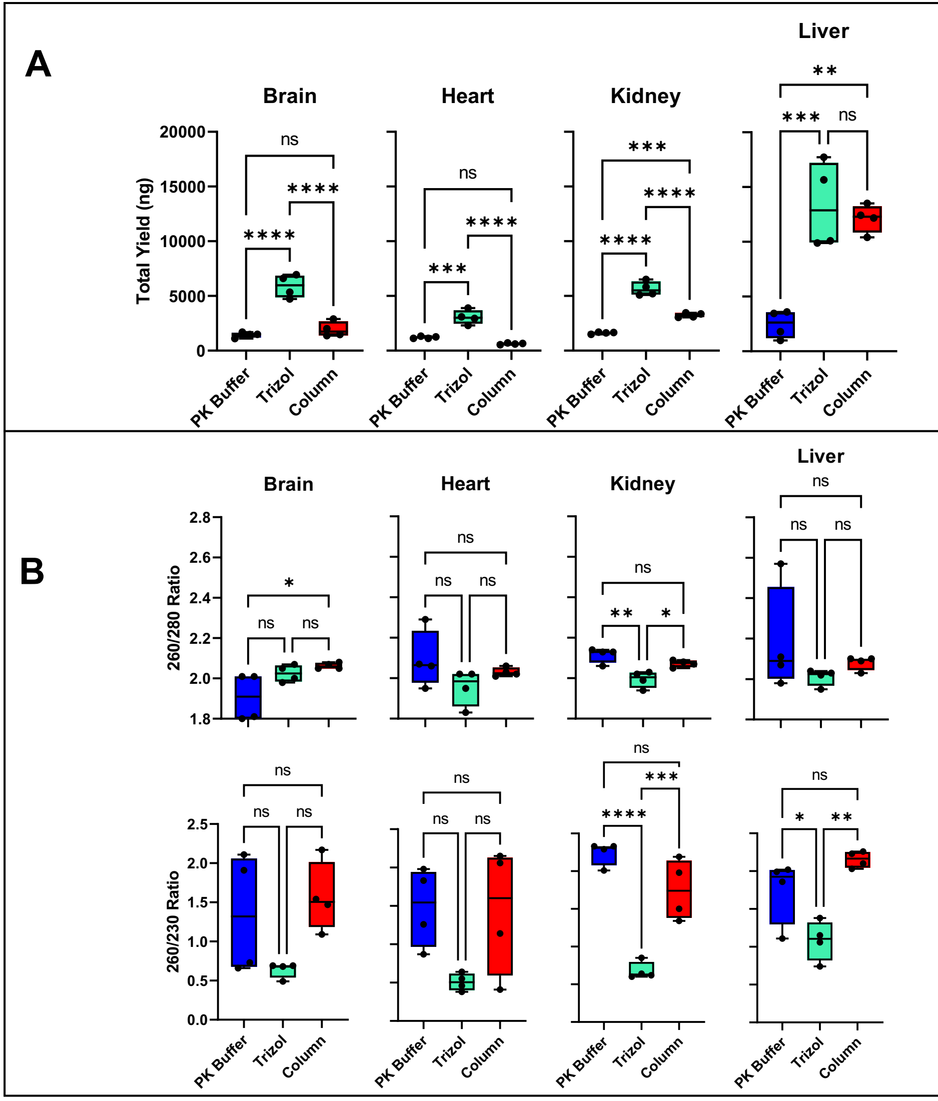

Total yield

Organs	Media	Mean (ng)	SEM
Brain	PK buffer	1437	61
Brain	TRizol	1206	47
Brain	PureLink	1620	44
Heart	PK buffer	2457	638
Heart	TRizol	5904	261
Heart	PureLink	3055	164
Kidney	PK buffer	5656	162
Kidney	TRizol	13345	989
Kidney	PureLink	1939	175
Liver	PK buffer	632	19
Liver	TRizol	3237	51
Liver	PureLink	12128	322

260/280

Organs	Media	Mean	SEM
Brain	PK buffer	2.01	0.00
Brain	TRizol	2.09	0.01
Brain	PureLink	2.06	0.01
Heart	PK buffer	2.00	0.02
Heart	TRizol	2.01	0.06
Heart	PureLink	1.99	0.05
Kidney	PK buffer	2.02	0.01
Kidney	TRizol	2.07	0.01
Kidney	PureLink	2.07	0.01
Liver	PK buffer	2.02	0.02
Liver	TRizol	1.97	0.02
Liver	PureLink	2.08	0.02

260/230

Organs	Media	Mean	SEM
Brain	PK buffer	1.84	0.10
Brain	TRizol	2.35	0.09
Brain	PureLink	1.57	0.22
Heart	PK buffer	1.91	0.03
Heart	TRizol	1.80	0.32
Heart	PureLink	1.12	0.49
Kidney	PK buffer	2.07	0.04
Kidney	TRizol	2.34	0.02
Kidney	PureLink	1.75	0.20
Liver	PK buffer	2.02	0.02
Liver	TRizol	2.13	0.14
Liver	PureLink	2.16	0.05

Fig.S4. Yields and quality of RNA isolated with PIXUL using PK buffer, TRIzol and PureLink column from mouse brain, heart, kidney and liver measured by NanoDrop. Frozen mouse organs in CryoTrays were sampled with the CryoCore and RNA was isolated using PIXUL with either PK buffer, TRIzol or PureLink column. ***A,*** RNA yields measured by NanoDrop. ***B,*** NanoDrop absorbance is shown as 260/280 (*upper row*) and 260/230 (*lower row*). Data are represented as box plots with whiskers indicating min/max and all data points overlaid, n=4 for each frozen organ. Statistical differences between methods (*P*-value) are shown by asterisks(*) between compared data; P≤0.05 by *, P≤0.01 by **, P≤0.001 by ***, P≤0.01 by ****, the "ns" indicating the differences are not statistically significant. Chart made with GraphPad Prism.

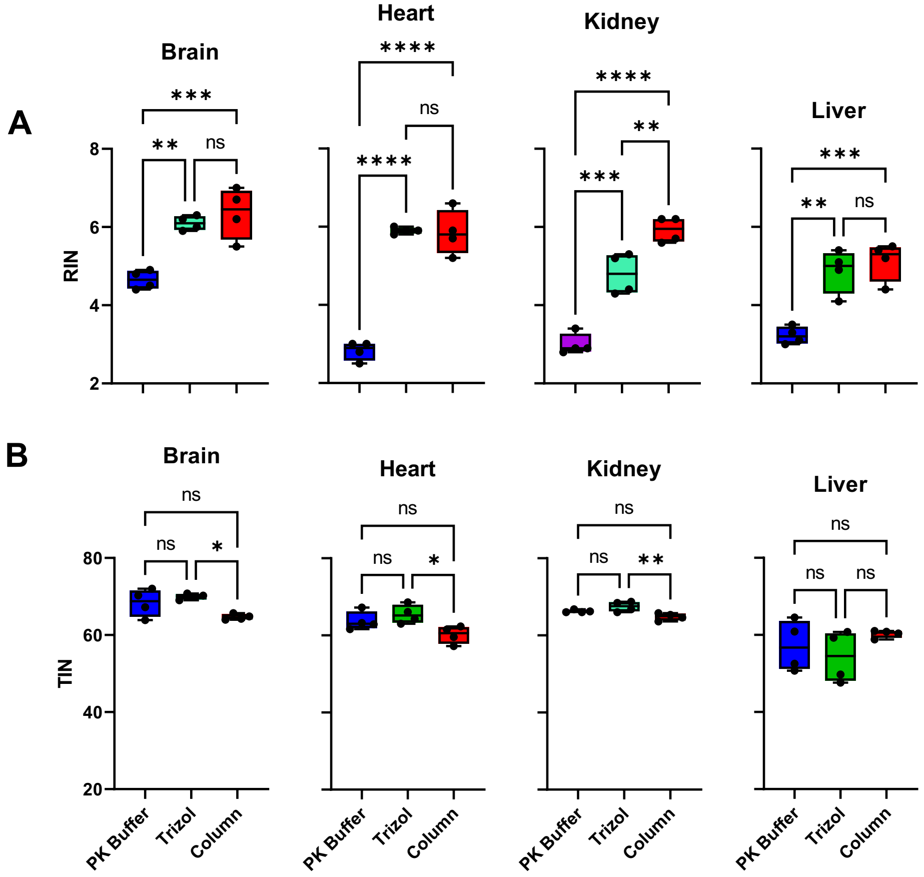

RIN

Organ	Media	Mean	SEM
Brain	PK buffer	4.71	0.15
Brain	TRIzol	5.83	0.14
Brain	PureLink	6.4	0.33
Heart	PK buffer	3.04	0.15
Heart	TRIzol	5.64	0.24
Heart	PureLink	5.9	0.29
Kidney	PK buffer	2.82	0.15
Kidney	TRIzol	4.13	0.15
Kidney	PureLink	6.1	0.13
Liver	PK buffer	3.04	0.13
Liver	TRIzol	4.73	0.21
Liver	PureLink	5.1	0.25

mTIN

Organ	Media	Mean	SEM
Brain	PK buffer	68.4	0.3
Brain	TRIzol	64.7	0.2
Brain	PureLink	70.0	0.3
Heart	PK buffer	63.7	1.1
Heart	TRIzol	60.2	0.7
Heart	PureLink	65.5	0.2
Kidney	PK buffer	66.3	0.1
Kidney	TRIzol	64.8	0.1
Kidney	PureLink	67.5	0.2
Liver	PK buffer	57.2	0.4
Liver	TRIzol	60.3	0.3
Liver	PureLink	54.4	0.7

Fig.S5. Sequencing quality assessed by RIN and mTIN. ***A***, High Sensitivity RNA ScreenTape® was used to assess the RNA integrity numbers (RIN) (50). ***B,*** Median Transcript Integrity Number (mTIN (28)) for each isolation method. Data are represented as box plots with whiskers indicating min/max and all data points overlaid, n=4 for each RNA isolation method. Statistical differences between methods (*P*-value) are shown by asterisks(*) between compared data; P≤0.05 by *, P≤0.01 by **, P≤0.001 by ***, P≤0.01 by ****, the "ns" indicating the differences are not statistically significant. Chart made with GraphPad Prism.

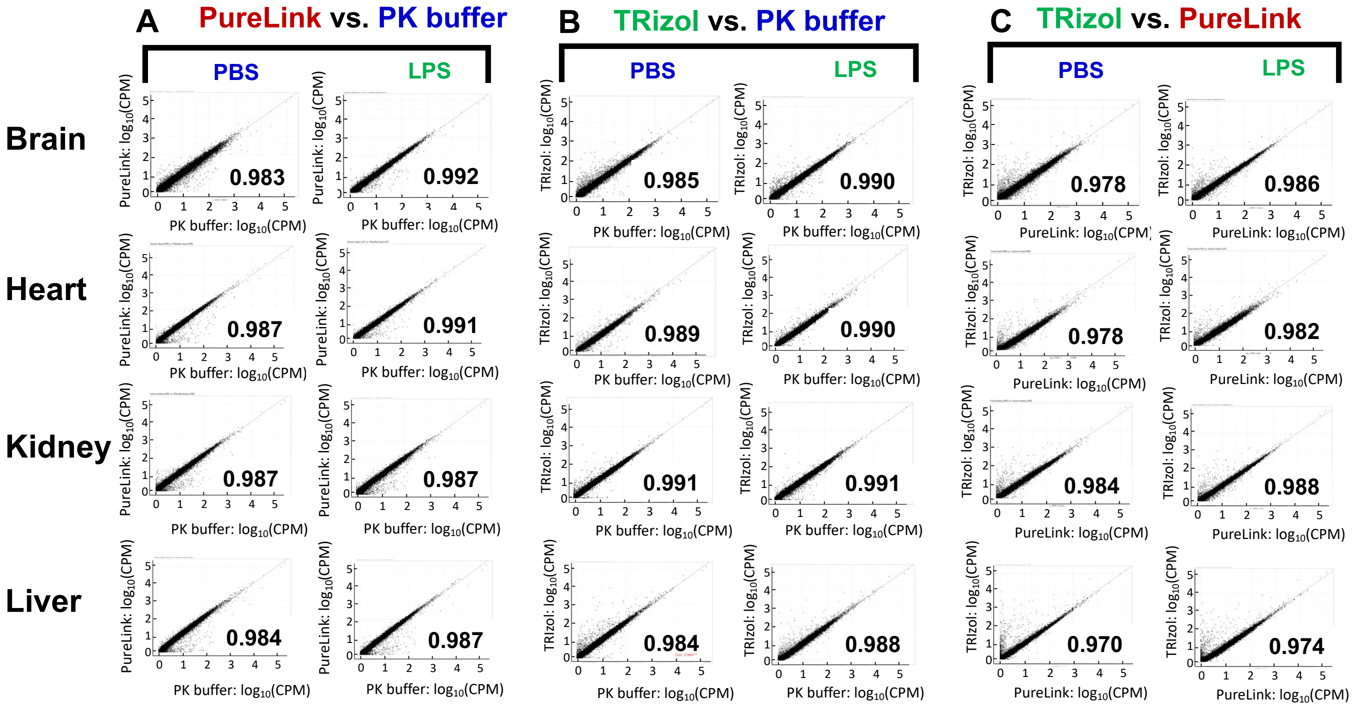

Fig.S6. Scatter plots of correlation analysis of PK buffer, TRIzol and PureLink columns of brain, heart, kidney and liver of RNA-seq normalized count data from frozen organs of septic (LPS IP injected) and control (PBS IP injected) mice. Results are shown as $\log_{10}$ counts per million reads mapped (CPM). ***A,*** *x*-axis PK buffer and *y*-axis PureLink column. ***B,*** *x*-axis PK buffer and *y*-axis Trizol ***C,*** *x*-axis PureLink columns and *y*-axis Trizol Numbers in the body of the graphs show correlation coefficients. Each dot represents a mean of two sets of frozen mouse organs.

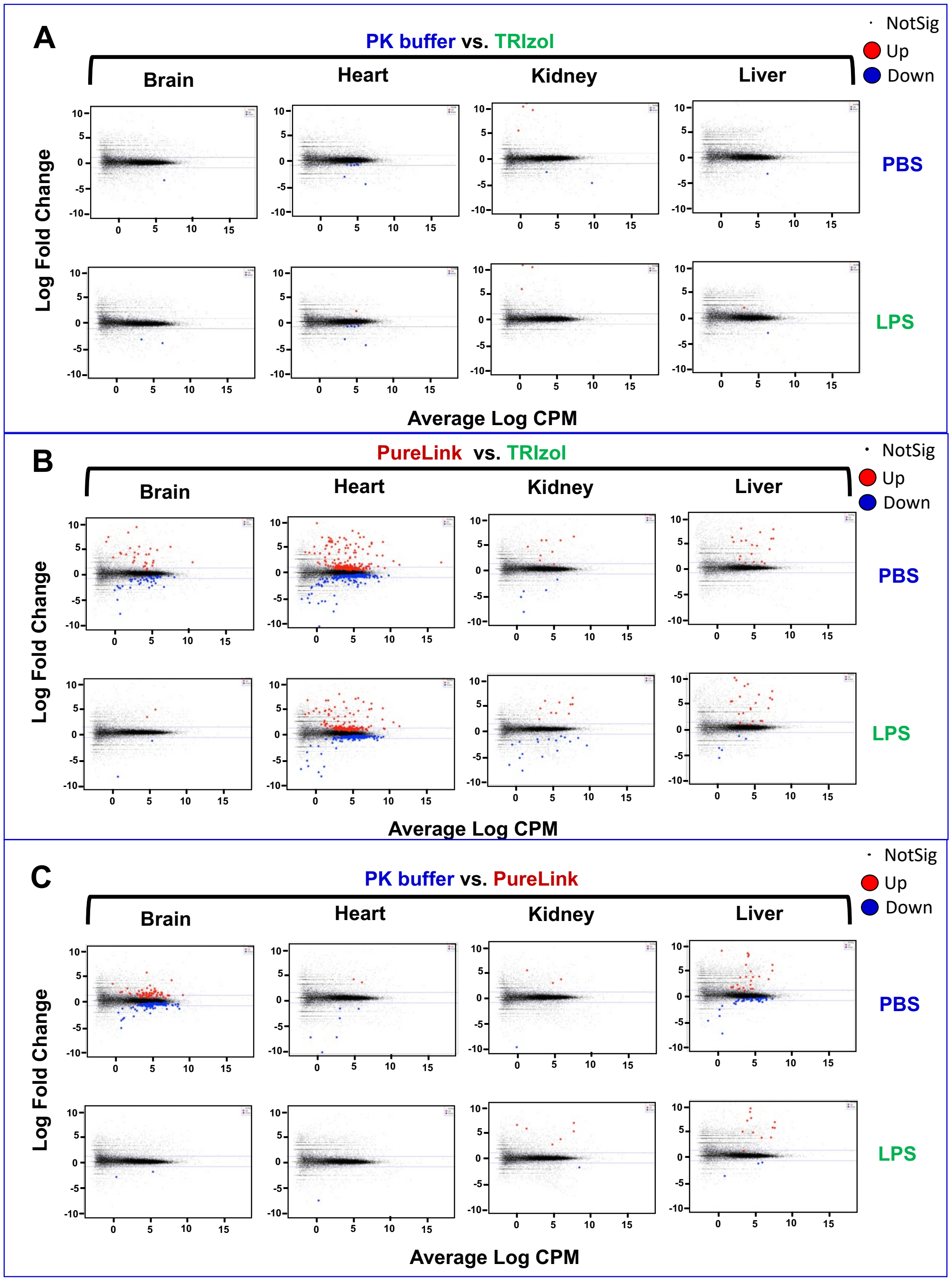

Fig.S7. 'EdgeR' Differential gene expression shown with mean-difference (MD) plots between the different RNA isolation methods. Comparison of brain, heart, kidney and liver transcripts from LPS treated compared to control (PBS) treated mice. MD plot shows log fold change, y-axis and average log of counts per million (CPM), x-axis. Red and blue dots indicate transcripts that are statistically increased or decreased, respectively. Black dots represent transcripts that were not significantly differentially expressed. ***A***, transcripts extracted with PK buffer compared to TRIzol from frozen brain, heart, kidney and liver from PBS- (upper panel) and LPS-treated mice. ***B***, transcripts extracted with PureLink column compared to TRIzol from frozen brain, heart, kidney and liver from PBS- (upper panel) and LPS-treated mice. ***C***, transcripts extracted with PK buffer compared to PureLink column from frozen brain, heart, kidney and liver from PBS- (upper panel) and LPS-treated mice.

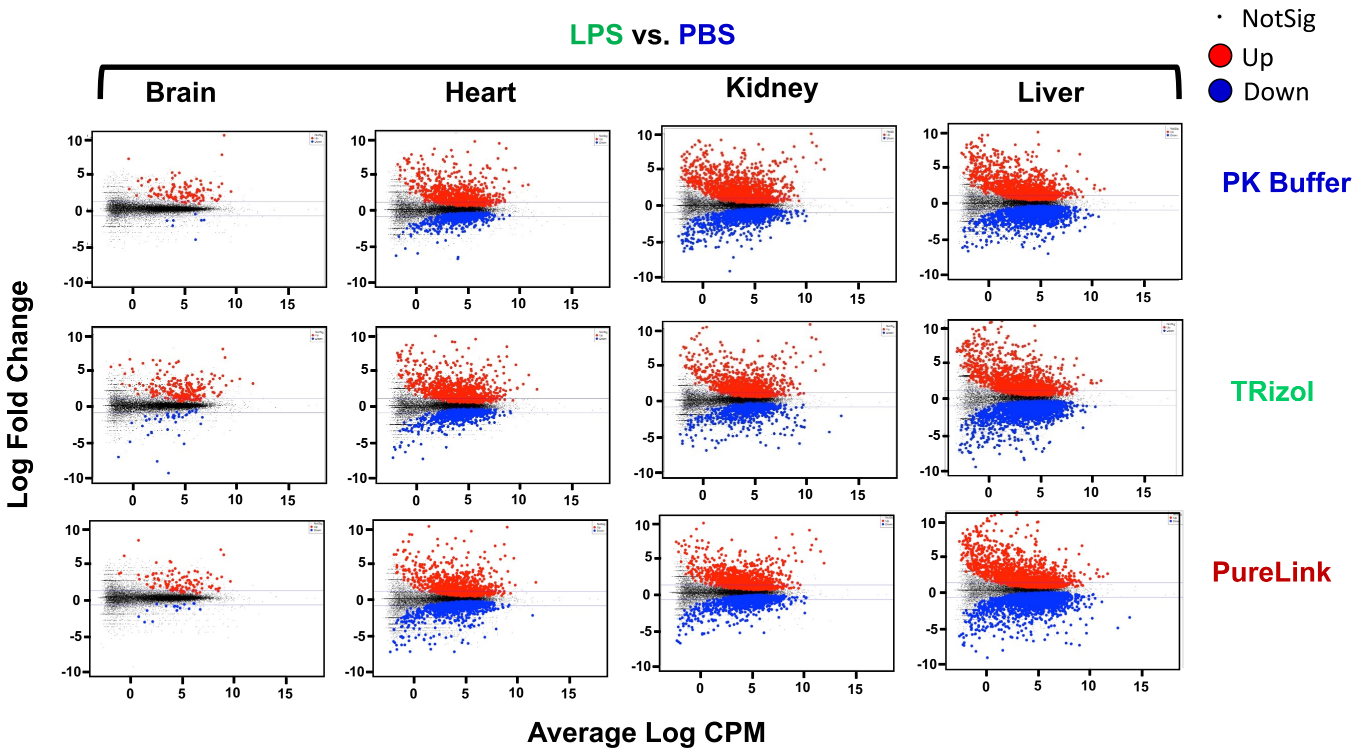

Fig.S8. 'EdgeR' Differential gene expression of LPS vs PBS treated mice shown with mean-difference (MD) plots for the different RNA isolation methods. Comparison of frozen brain, heart, kidney and liver transcripts from LPS-treated compared to PBS-treated (control) mice, *upper panel* PK buffer, *middle panel* TRIzol and *lower panel* PureLink column extracted RNA, respectively. y-axis log fold change; x-axis average log CPM.

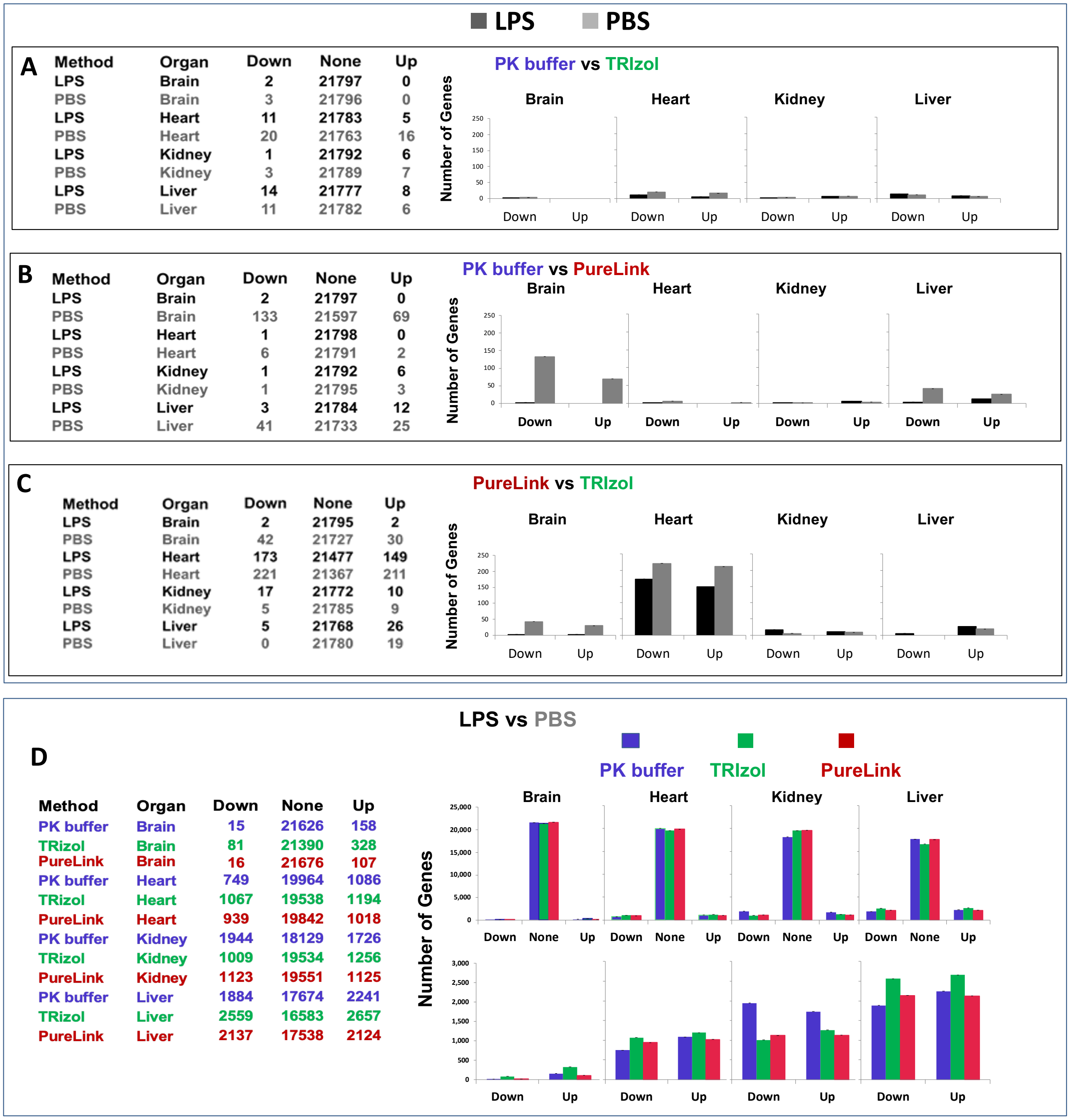

A

Method	Organ	Down	None	Up
LPS	Brain	2	21797	0
PBS	Brain	3	21796	0
LPS	Heart	11	21783	5
PBS	Heart	20	21763	16
LPS	Kidney	1	21792	6
PBS	Kidney	3	21789	7
LPS	Liver	14	21777	8
PBS	Liver	11	21782	6

B

Method	Organ	Down	None	Up
LPS	Brain	2	21797	0
PBS	Brain	133	21597	69
LPS	Heart	1	21798	0
PBS	Heart	6	21791	2
LPS	Kidney	1	21792	6
PBS	Kidney	1	21795	3
LPS	Liver	3	21784	12
PBS	Liver	41	21733	25

C

Method	Organ	Down	None	Up
LPS	Brain	2	21795	2
PBS	Brain	42	21727	30
LPS	Heart	173	21477	149
PBS	Heart	221	21367	211
LPS	Kidney	17	21772	10
PBS	Kidney	5	21785	9
LPS	Liver	5	21768	26
PBS	Liver	0	21780	19

D

Method	Organ	Down	None	Up
PK buffer	Brain	15	21626	158
TRIzol	Brain	81	21390	328
PureLink	Brain	16	21676	107
PK buffer	Heart	749	19964	1086
TRIzol	Heart	1067	19538	1194
PureLink	Heart	939	19842	1018
PK buffer	Kidney	1944	18129	1726
TRIzol	Kidney	1009	19534	1256
PureLink	Kidney	1123	19551	1125
PK buffer	Liver	1884	17674	2241
TRIzol	Liver	2559	16583	2657
PureLink	Liver	2137	17538	2124

Fig.S9. RNA-seq data of differentially expressed genes in LPS endotoxin and PBS (control) treated mice. ***A-C,*** numbers of transcripts that were different in frozen organs from LPS and PBS treated mice comparing PK buffer vs. TRIzol (A), PK buffer vs. PureLink (B) and PureLink vs. TRIzol (C). ***D***, PK buffer, TRIzol and PureLink columns comparison. Table shows the number of genes data for each group used to generate plots on the *right*. *Right upper* panels show comparison of the number of frozen organ transcripts extracted with either PK buffer, TRIzol or PureLink columns from LPS compared to PBS treated mice that were upregulated (*Up*), downregulated (*Down*) or were not changed (*None*). To better show the differences the *right lower* panels shows comparison of the number of organ transcripts extracted with either PK buffer, TRIzol or PureLink column from LPS compared to PBS treated mice that were upregulated (*Up*) or downregulated (*Down*).

TABLES

Table S1. Hardware and labware

Item Description	Manufacturer/Supplier	Location	Part No.
PIXUL™ 96-well sonicator	Matchstick Technologies	Kirkland, WA	P01199-01A
	Active Motif	Carlsbad, CA	53130
CryoTray	Matchstick Technologies	Kirkland, WA	P01200-01A
CryoBlock	Matchstick Technologies	Kirkland, WA	P01201-01A
CryoCore	Matchstick Technologies	Kirkland, WA	P01202-01A
CryoCore trephines	Matchstick Technologies	Kirkland, WA	P01203-01A
PlateHandle	Matchstick Technologies	Kirkland, WA	P01204-01A
Fluke 52-II Dual Probe Thermometer	Fluke Corporation	Everett, WA	674689
Fluke 80PJ-1 Bead Probe	Fluke Corporation	Everett, WA	750422
Small Dual Angle Beta Radiation Shield (used as iPAD stand)	Universal Medical Inc.	Oldsmar, FL	UM3400
iPAD Pro	Apple	Cupertino, CA	Model A2377
NextSeq2000	Illumina	San Diego, CA	20038897
4200 TapeStation	Agilent	Santa Clara, CA	G29991BA
Invitrogen™ Qubit™ 3 Fluorometer	ThermoFisher	Waltham, MA	Q33216
NanoDrop 1000 Spectrophotometer	ThermoFisher	Waltham, MA	
Eppendorf ThermoMixer® C	Eppendorf	Hamburg, Germany	5382000023
Costar 96-well polypropylene round-bottom (used in PIXUL)	Corning	Oneonta, NY	3365
GeneMate 96-well 0.2ml plate semi skirt	VWR	Radnor, PA	490003-794
MicroAmp Optical Adhesive Film (used in PIXUL)	Applied Biosystems	Carlsbad, CA	4311971
E1-ClipTip™ Electronic Multichannel Pipettes	ThermoFisher	Waltham, MA	4671090
7900HT Fast Real-Time PCR System with 384-Well Block Module	ThermoFisher	Waltham, MA	4329001

Table S2. Kits and enzymes

Item Description	Manufacturer/Supplier	Location	Part No.
Zymo-Seq RiboFree Total RNA Library Kit	Zymo Research	Tustin, CA	R3003
Zymo RNA Clean & Concentrator-5	Zymo Research	Tustin, CA	R1016
PureLink™ RNA Micro Kit	Invitrogen	Carlsbad, CA	12183016
SpeedBeads™ magnetic carboxylate modified particles	MilliporeSigma	Burlington, MA	GE65152105050250
SuperScript IV Reverse Transcriptase	ThermoFisher	Waltham, MA	18090050
RNaseOUT™ Recombinant Ribonuclease Inhibitor	ThermoFisher	Waltham, MA	10777-019
RNase-Free DNase I	Lucigen	Middleton, WI	E0013-1D4
DNase I 10X Reaction Buffer	Lucigen	Middleton, WI	SS000751-D2
Invitrogen™ Proteinase K, recombinant-Invitrogen	ThermoFisher	Waltham, MA	25530015
Standard Sensitivity Large Fragment Analysis Kit	Agilent	Santa Clara, CA	DNF-492
High Sensitivity RNA ScreenTape Ladder	Agilent	Santa Clara, CA	5067-5581
High Sensitivity RNA ScreenTape Sample Buffer	Agilent	Santa Clara, CA	5067-5580
High Sensitivity RNA ScreenTape	Agilent	Santa Clara, CA	5067-5579
NextSeq™1000/2000 P2	Illumina	San Diego, CA	20046811
Collibri™ Library Quantification Kit	Invitrogen	Carlsbad, CA	A38524500
Quant-it dsDNA High-Sensitivity Assay Kit (Qubit)	Invitrogen	Carlsbad, CA	Q33120

Table. S3. qPCR primers

Species	Gene	Purpose	Forward	Reverse
Mouse	*Rlp32* Exon2	RT	TTAAGCGAAACTGGCGGAAAC	TTGTTGCTCCCATAACCGATG
Mouse	*Actb* Exon3-Exon4	RT	CTAAGGCCAACCGTGAAAAG	GCATACAGGGACAGCACA
Mouse	*Alb* Exon 15	RT	TGAAGACTCAGGACTCATCTTTT	CAGCACAGAGACAAGAAGTC
Mouse	*Fxyd2* Exon 6	RT	CAGCTTCTCTAACACCCCAC	GATTCATTGAAAACCAGGGGG
Mouse	*Tnnt2* Exon15	RT	CCAATGCAGACTCCTGTTTG	TGGCTTTTTATTGCTGGCAT
Mouse	*Ngal* Exon5-Exon6	RT	TCAAGGACGACAACATCATCT	CACTCACCACCCATTCAGTT
Mouse	*Syn 1* Exon13	RT	AGGTGAAAGCTGAGACCATC	TGTCTAGGGGTTTAGGGGTT
Human	*RLP32* Exon2-Exon3	RT	AGTTCCTGGTCCACAACGTC	TTGGGGTTGGTGACTCTGAT
Human	*ACTB* Exon3-Exon4	RT	AGAGCTACGAGCTGCCTGAC	AAGGTAGTTTCGTGGATGCC
Human	*EGR1* Exon1-Exon2	RT	TGACCGCAGAGTCTTTTCCT	TGGTTTGGCTGGGGTAACT
Human	*EGR1* Exon2	RT	GCTGAGCTGAGCTTCGGTTC	TCGCCGCCTACTCAGTAGGTA
Human	*NR4A1* Exon7	RT	TGACCCCACGATTTGTCTTA	ACGGCGCTATGTGTTAA
Human	*NR4A1* Exon7-Exon8	RT	CACAGCTTGCTTGTCGATGT	CATGCCGGTCGGTGATGA
